# Automating Diagnosis of Skin Neglected Tropical Diseases via Patient Metadata through Machine Learning Model with Adaptive Balancing and Dual Cross-Validation: Retrospective Diagnostic Accuracy Study

**DOI:** 10.2196/84966

**Published:** 2026-08-21

**Authors:** Yohannes Minyilu, Mohammed Abebe Yimer, Million Meshesha

**Affiliations:** 1 Faculty of Computing and Software Engineering, Arba Minch Institute of Technology, Arba Minch University, P.O. Box 21, Arba Minch, Ethiopia, +251 911434681; 2 School of Information Science, College of Natural and Computational Sciences, Addis Ababa University, Addis Ababa, Ethiopia

**Keywords:** skin NTDs, patient metadata, ML models, feature importance, class weighting, dual cross-validation, machine learning, neglected tropical diseases

## Abstract

**Background:**

Skin neglected tropical diseases (NTDs) are the most prevalent diseases worldwide, affecting people living in resource-limited areas with low health care services and trained professionals. While machine learning (ML)–based diagnostic tools can be used for initial clinical assessment and patient screening, especially in resource-limited areas (including in Ethiopia), little effort has been made in this area.

**Objective:**

This pilot study develops a foundational ML model for the diagnosis of skin NTDs using patient metadata to analyze the feasibility of ML-based models for skin NTDs by identifying and experimentally evaluating 8 ML models.

**Methods:**

For this study, we acquired a tabular skin NTD diagnostic dataset collected from a specific affected district in the southwest of Ethiopia. We used the data in 3 different structures, which include using the initial dataset (IDS) that contains huge null values, using a final dataset (FDS) created through preliminary preprocessing, and a third dataset created by applying feature engineering (FEFDS). Selecting 8 ML models, we trained the models in 4 major experimental settings: baseline training, handling structural missing values, handling severe class imbalance through conditional class weighting, and a hybrid approach based on robust dual cross-validation (CV) consisting of an outer repeated stratified *k*-fold and nested CV methods. We used the macro and class-specific metrics (such as precision, recall, and *F*_1_-score), including balanced accuracy, due to the severe class imbalance. Feature importance scores are also used for evaluating overall model performance.

**Results:**

After the final training applying the hybrid approach, 4 models scored a perfect test score (1.0) across all the metrics and all experiments except naïve Bayes and multilayer perceptron (MLP), similarly scoring 0.997 balanced accuracy, 0.97 recall, and 0.985 *F*_1_-score. In the same experiment, the nested CV loop revealed a slight performance drop for light gradient boosting machine (LightGBM) and extreme gradient boosting (XGBoost), though both models similarly maintained higher mean scores of balanced accuracy and macro recall of 0.993 (SD 0.013), including the mean macro *F*_1_-score of 0.996 (SD 0.007), scoring declining performance with a mean score of 0.993 (SD 0.013) in balanced accuracy, macro recall, and *F*_1_-score, showing predictive biases. In terms of feature utilization, only CatBoost showed optimal feature ranking, while 4 models showed over-feature utilization (selection bias), with 3 models using small subsets of features (showing feature parsimony).

**Conclusions:**

Overall, this study has been highly challenged by data scarcity, class imbalances, limited disease representation, specific geographic representation, and lack of more data modalities. Hence, further studies are suggested to confirm the results on larger datasets having a representative distribution of disease classes and geographic locations.

## Introduction

### Background

Neglected tropical diseases (NTDs) are the most prevalent diseases worldwide. According to the World Health Organization (WHO), NTDs represent about 20 different diseases, such as podoconiosis, scabies, tungiasis, buruli ulcer, cutaneous leishmaniasis (CL), leprosy, mycetoma, and rabies [1-3]. Ten of the NTDs, according to WHO, are known to exhibit primary skin symptoms (hence skin NTDs) or related clinical symptoms that often can cause bodily harm and disfigurement, which can cause disability [4]. As a tropical country, the majority of NTDs are present in Ethiopia [5], particularly in the remote areas of the country. Recent figures showed that Ethiopia remains one of the countries with a high burden of NTDs, where more than 75 million people are at risk of contracting at least one of the NTDs [6,7].

Clinically, different diagnostic procedures are used for the diagnosis of skin NTDs, where the dermatological approach is the primary method used. Marks et al [8], on behalf of the WHO Diagnostic Technical Advisory Group (DTAG), highlighted that the integration of clinical and laboratory diagnostic services is the fundamental approach for skin NTDs, where initial clinical assessment of patients, followed by confirmatory tests (laboratory diagnostics), are the 2 critical steps toward enhanced diagnostics of skin NTDs. However, establishment of laboratory facilities in most NTD-vulnerable areas (based on current real-world scenarios) is hardly feasible due to higher resource requirements. Such scenarios guide efforts to look for alternative, technology-assisted solutions through decentralized diagnostic frameworks. Tadesse et al [9], who studied the decentralized diagnosis of CL in Ethiopia, claimed that with minor adjustments to health care facilities and continuous training of primary health care professionals, the diagnosis and treatment of skin NTDs can be decentralized to the health care center levels.

Furthermore, recent studies such as [6,10] suggested the feasibility of mobile-based diagnostic platforms for resource-limited settings. The authors in [11] and [12] used advanced AI-based diagnostic tools based on machine learning (ML) and deep learning (DL) methods for the diagnosis of skin NTDs. The use of such tools would facilitate the creation of localized diagnostic platforms, as they provide the opportunity for patients to get health care services around their living areas, especially in resource-limited areas. This triggers the need to investigate and implement NTD diagnostics based on effective point-of-care tools for skin NTD diagnosis [8,9,13]. Accordingly, ML-based diagnostic tools based on clinical patient data can be used for initial clinical assessment of patients and patient screening, as well as patient record organization and management, especially in resource-limited areas. Most importantly, in resource-constrained areas, ML-based diagnostic tools implemented based on the differential diagnostic approach can be used by middle-level health care workers for early diagnosis of patients with skin NTDs. Therefore, this study investigates the feasibility of an ML-based skin NTD diagnostic framework by proposing an ML-based diagnostic model for skin NTDs using patient metadata. Accordingly, the study is designed to be a pilot study that intends to establish the foundational basis for the realization of an intelligent diagnostic model for skin NTDs.

In this pilot study, we establish a robust and integrated framework for the systematic investigation of the feasibility of high-performing diagnostic ML models for skin NTDs based on tabular clinical data. This includes establishing integrated pipelines for data preprocessing, handling missing data, and addressing class imbalance, including the identification, synthesis, and evaluation of ML architectures. For this study, we used a novel dataset created using newly collected tabular patient metadata of 3 skin NTDs (podoconiosis, scabies, and tungiasis) to train and evaluate 8 selected ML models. We further conducted a comparative performance analysis of the trained models to identify the model with the highest performance and reliability, as well as the optimal ML strategy that resulted in the best performance. The study also analyzed the impact of missing values and strategies for handling missing data (imputation methods), which serves as a benchmark for further studies. However, as the study presents a foundational framework, it does not intend to build a full-fledged and deployment-ready diagnostic model; as such, complete systems require sufficiently large datasets, high computational resources, complex and multistaged evaluation, and regulatory procedures before deployment.

Overall, apart from being the first study that proposes the application of ML-based diagnostics for skin NTDs using tabular data, this study presents other novel contributions, which include presenting a novel tabular skin NTD dataset and the establishment of a harmonized ML pipeline. Accordingly, we identified and used a set of ML strategies and created a robust ML model development pipeline for the systematic application, evaluation, and analysis of methods and models, which include comprehensive baseline training setup, rigorous feature ranking and importance analysis, robust implementation of missing data handling, and the harmonized approach—the robust ML pipeline that synergistically integrates conditional class weighting with a dual CV evaluation approach based on the integration of 2 CV methods (repeated and nested CV methods). The collective implementation of all these strategies helps in establishing an overall robust ML pipeline, clearly marking the novelty of our study.

### Related Works

#### Machine Learning Models for Structured Data

Developing ML-based diagnostic tools requires carefully devised strategies for the selection, utilization, evaluation, and optimization of the ML models and tools. Accordingly, the type of data to be used, computational resources available for model building and deployment, expected model performances, undesired model behaviors such as overfitting, model optimization strategies to be used, and the nature of the disease to be diagnosed are the major parameters that determine the ML methods and strategies to be used. For small-sized datasets, support vector machines (SVMs), decision trees, and random forests (RF) are the preferred ML algorithms, while the convolutional neural network (CNN)–based DL models can also be used [14,15]. However, the tree-based model extreme gradient boosting (XGBoost) is the most suitable model for tabular data since it requires fewer hyperparameter tuning tasks compared to the DL-based models, as stated in [16]. Generally, the gradient-boosted tree models of XGBoost, light gradient boosting machine (LightGBM), and CatBoost are well-suited for tabular data, although they can be challenged by model complexity [17], higher computational resource requirements such as GPUs for large datasets [18], and possible issues during real-time deployment on edge devices [19].

#### Machine Learning Methods for Data-Related Constraints

There are multiple ML strategies and models for handling dataset-related issues, as it is sometimes challenging to acquire complete datasets that are sufficient to train ML and DL models. For small-sized tabular datasets, traditional ML models and ensemble approaches are the most suitable methods due to their stability and interpretability, as indicated in recent studies. Accordingly, the tree-based ensemble models of RF and extra trees are most often the top-performing models for the classification of diseases in dermatology based on tabular patient metadata, including the situation of having small-sized tabular datasets [20]. In terms of performance reliability, *k*-nearest neighbor (KNN) and SVM are the most stable models that resulted in superior performance compared to complex neural network–based models for small-sized structured clinical datasets [21,22]. While the boosted tree-based model XGBoost requires a larger number of samples to reach performance stability, the RF model appears to be a more reliable model for small-sized datasets [22]. Regarding missing data, studies suggested that the gradient-boosted tree models of CatBoost, XGBoost, and LightGBM are highly robust to missing data when trained on incomplete patient records. Accordingly, CatBoost (with nan_mode enabled) is the most robust model, as it has an internal mechanism to treat “NaN” values as a separate split direction [23]. The skin disease detection study by Aquil et al [20] highlighted that XGBoost and LightGBM models have the ability of learning “default direction” for missing values during training. Accordingly, if a given symptom (feature) has a null value, the model automatically assigns this feature to the branch that minimizes the loss based on other patients.

#### Machine Learning Methods for Skin NTD Diagnosis

Most previous studies conducted for the diagnosis of skin NTDs heavily rely on skin images. Accordingly, the previous studies [23-25] used DL-based methods to apply diagnostic models for skin NTDs, where all these studies used only skin image-based approaches. On the other hand, previous works that implemented ML methods for the diagnosis of skin NTDs using only structured clinical data were difficult to find, except 2 studies that used tabular patient metadata along with skin image data. Accordingly, the study by Barbieri et al [11] used structured clinical data of leprosy patients to integrate with skin lesion images and used ML algorithms that include RF and XGBoost for handling the clinical patient data in their multimodal data fusion study. Similarly, Achary et al [12] used tabular clinical metadata to apply multimodal data fusion by combining the metadata with skin images of patients using generative models for text data augmentation. In both studies, the tabular clinical data of patients are not used independently for skin NTD diagnosis; instead, they are merged with image data based on multimodal data fusion methods.

Overall, the results of the literature review underscored that structured clinical metadata of patients are not fully used, let alone the independent utilization, demonstrating the development of ML-based diagnostic models for skin NTDs based on tabular clinical patient data, indicating a clear research gap. One of the biggest challenges in building AI-based diagnostic tools for skin NTDs is the lack of large-scale, complete, and properly annotated datasets, as affirmed by the study by Achary et al [12]—the study used generative models to generate textual data to fill the gap of data scarcity. Conversely, the selection and use of specific ML strategies require further investigation, given that skin NTD data are naturally scarce resources, including limited previous studies for comparative analysis of methods. All these factors clearly mark research-wide gaps that forced the authors of this study to conduct this study.

## Methods

### Study Design

This pilot study develops a foundational benchmark diagnostic model for skin NTDs using structured (tabular) clinical patient data by selecting the best-performing model from a set of 8 ML models initially selected for the experiments. For a complete and fair comparison, all models are equally trained on similar datasets, using similar training settings. To achieve this objective, the study relies on capturing and analyzing model performance results by conducting a series of model training experiments. This highlights both the qualitative and quantitative aspects based on the experimental approach. Therefore, this study uses a mixed research strategy. However, this study does not currently intend to realize a fully deployable diagnostic model.

### Ethical Considerations

This study uses a novel dataset created by acquiring clinical records consisting of demographic information and disease-specific diagnostic data of patients with skin NTDs. The entire data collection and acquisition processes are carried out in a professional and ethical manner, which include acquiring an ethical clearance letter that authorizes the data collection and use of the collected data for this study, and confirming all legal and ethical issues, including written consent of patients. To achieve this, the authors of this study have acquired a proper ethical clearance letter from the Institutional Review Board committee at Arba Minch University (approval protocol number: YM23161).

### Data Acquisition and Dataset Preparation

#### Data Source

For this study, we created a new hand-crafted dataset using the data collected from patients with skin NTDs living in Gacho Baba district of the Gamo zone, southwest of Ethiopia, which is one of the remote areas representing underserved communities in terms of health care services. The data were collected by a team of researchers from the Collaborative Research and Training Center for NTDs, College of Medicine and Health Sciences of Arba Minch University, in a project-based research conducted for the assessment of skin NTD burden. For this study, the original (first-hand) data were acquired through institutional collaboration (acknowledgments section) and used to achieve the objective of this study.

#### Initial Diagnostics and Confirmation

The entire data collection was initially conducted in a large-scale community-based mass drug administration (MDA) campaign in the specified community based on on-site clinical diagnostic procedures. At the MDA site, however, extended medical resources like laboratory facilities, specialized medical imaging, or any advanced medical infrastructure were not available. In such settings, the initial clinical diagnostic procedures were performed by trained frontline health care workers (mostly middle-level clinicians and nurses) who were actively involved in the day-to-day clinical diagnostics of patients around the area, and a professional dermatologist from Arba Minch General Hospital who participated during the MDA campaign. Therefore, the on-site diagnostics were performed entirely based on visual clinical examination procedures by the frontline clinicians, with strict diagnostic confirmation by the professional dermatologist.

As the data collection involved actual field-based diagnoses, the establishment of initial ground truth was challenged by limitations related to field logistics. To overcome this challenge, a hierarchical diagnostic verification protocol was applied by the data collection team to clinically confirm the diagnostics. Hence, each of the initial diagnostic procedures underwent a mandatory clinical review by a professional dermatologist from Arba Minch General Hospital to confirm the initial diagnostics. While this procedure confirms the correct classification of the skin NTD diagnosed based on the signs and symptoms presented by the patients, it also helped in creating higher-level diagnostic fidelity. Overall, the ground truth we used for the classification of the skin NTDs for this study complies with the WHO-standardized clinical case definitions [26]. After all these clinical and ethical procedures were completed, the authors of this study used the clinically validated data for the ML-based research in this study after acquiring the proper authorization through ethical clearance.

#### Data Collection

Overall, the data collection was conducted based on a community-based screening approach for assessing the burden of skin NTDs, which included on-site examination, treatment, and registration of patient information. To achieve this, the data collection team used a well-prepared data collection and patient registration form, which was prepared by consulting professional dermatologists from Arba Minch General Hospital. The data collection involved 4029 participants during the initial screening and diagnosis, targeting the identification and diagnosis of 8 skin NTDs that are endemic to Ethiopia, which include mycetoma, leprosy, CL, lymphatic filariasis, podoconiosis (podo), onchocerciasis, tungiasis, and head lice. Finally, according to the data collection report, the specified community was highly affected by 3 of the skin NTDs, namely scabies, tungiasis, and podoconiosis. Thus, we selected these 3 diseases for our study based on the prevalence and availability of sufficient data. The other 5 mentioned diseases have very little (or no data at all) in the specified community at the time of data collection; hence, they are not used in the final dataset.

#### Limitations of the Data Collection

Generally, collecting large-scale skin NTD data from wider geographic areas (like Ethiopia) with complete representation of all endemic diseases is a complex and highly resource-intensive process, which is almost impossible to achieve in a single research project. Hence, our study is limited to a single geographic location in the Gacho Baba district, southwest of Ethiopia. This confinement to the specific location limited the number of endemic diseases to only 3 skin NTDs (scabies, tungiasis, and podoconiosis) out of the 8 overall endemic diseases, which forced us to exclude other skin NTDs like buruli ulcer, leishmaniasis, leprosy, and mycetoma. However, as a preliminary pilot study, we used the acquired skin NTD data to build a benchmark skin NTD diagnostic model, with the potential of extending the disease representation through further efforts.

#### Dataset Creation

During initial collection, the patient data collected for each of the 3 diseases were recorded separately using individual Microsoft Excel files for each disease. Hence, we initially created 3 separate datasets, which represent (1) scabies, which consists of 955 instances having 10 features (6 demographic and 4 disease-specific); (2) tungiasis, consisting of 474 instances having 8 features (6 demographic and 2 disease-specific); and (3) podoconiosis, comprising 66 instances having 11 features (6 demographic and 5 disease-specific), all before data preprocessing. In each of these 3 separate datasets, all disease-specific features (symptoms) are represented in binary categorical form using either “1” or “0” to signify the presence or absence of a given disease-specific symptom in a patient, which were applied after clinical confirmation of all diagnoses. For instance, “slowenlarging_swelling(feet&legs)” is a symptom specifically assessed while diagnosing podoconiosis, and all 66 podo patients were assessed if they manifest this particular symptom. Hence, after final clinical confirmation of the diagnosis, this feature is encoded as either “1” if a given patient showed this symptom or “0” otherwise, where a similar pattern was used for all symptoms of the 3 diseases.

Finally, we handcrafted our initial dataset (named it IDS) by merging the 3 individual disease-specific datasets. Originally, IDS comprises a total of 1495 instances with 17 trainable features (6 demographic and 11 diagnostic) and the target variable “disease_diagnosed” defining the class labels. After initial data preprocessing, IDS is transformed into a new structure having 1495 instances with 28 trainable features (17 demographic and 11 diagnostic) plus the target variable. However, the merger of the 3 separate disease-specific datasets introduced a different challenge due to the disease-specific symptoms, as not all symptoms are applicable to all diseases—a specific symptom that can be used to diagnose scabies is not applicable to the other 2 diseases. This created 3 blocks of missing values in IDS, showing a structural missingness issue [27]. Hence, using ML-based operations, we created 2 additional datasets through transformation: first, we created a final dataset (FDS), having 28 trainable features (17 demographic and 11 disease-specific features); next, we created a different dataset by applying feature engineering on the final dataset (FEFDS), having 38 trainable features (17 demographic and 11 disease-specific features, including 10 symptom applicability indicator flags introduced after feature engineering). We created all these 3 different datasets in 3 separate CSV files to address structural missingness, ensure reproducibility, and maintain dataset integrity across different experimental settings, thereby allowing us to establish a consistent training and evaluation pipeline.

### Exploratory Data Analysis

#### Dataset Characteristics and Missingness Analysis

Multimedia Appendix 1 presents the overall distribution of instances (n=1495), disease classes, and missing values in IDS. As shown in Figure S1 of Multimedia Appendix 1, the scabies disease class represents the largest proportion with a total of 955 (63.9%) instances, while tungiasis represents the second largest proportion with 474 (31.7%) instances, and podoconiosis has only 66 (4.4%) instances. Gender-wise, 66.6% (995/1495) of the patients are male, while 33.4% (500/1495) are female. These statistics clearly highlight that the dataset exhibits a severe class imbalance among the 3 disease classes, having an extremely distorted distribution with an overall imbalance ratio of 14.5 between scabies (majority class) and podoconiosis (minority class).

Correspondingly, Figure S2 in Multimedia Appendix 1 depicts the statistical summary of missing values in the dataset. As stated, our new dataset is created by merging 3 separate disease-specific datasets, each of which has a unique set of clinically relevant symptoms that are not applicable to the other diseases. For instance, the symptom “PPSEs” (which represents “papule, pustule, scratches, and excoriations”) is assessed only for scabies patients, where this field is structurally empty for podoconiosis and tungiasis patients. Ultimately, this created 3 blocks of missing values that define the case of structural missingness or missing by design [27], where our new dataset contains a total of 11,347 such structural null values. The missing values in our dataset are not random omissions created as a result of the random absence or exclusion of symptoms that are called “missing completely at random (MCAR)” or “missing at random (MAR)” [28]. Overall, as depicted in Multimedia Appendix 1, the scabies class has the highest number of null values, with 58.9% (6685/11,347) of the total number of null values (having 7 null values per each of the 955 rows), while tungiasis has 37.6% (4266/11,347), and podoconiosis has 3.5% (396/11,347) since it has the least number of instances.

#### Feature Description

Table 1 presents the overall summary of our first dataset, IDS, including its overall statistical summary, including its overall statistical summary of class distribution, feature categories, and the initial feature sets of IDS.

As shown, our new dataset (IDS) primarily contains two major categories of patient data: (1) demographics, which include 2 numerical features (age and weight) and 3 categorical features (gender, marital status, educational level, and occupation) of the patient information, and (2) clinical data, which comprises 11 disease-specific symptoms from the 3 classes. Class-wise, podoconiosis has 5 specific features (disease-specific symptoms) that are not applicable to the other 2 diseases, and the scabies class has 4 unique symptoms applicable only to scabies, while there are only 2 tungiasis-specific symptoms (features). Overall, our dataset has 2 features representing numerical data (age and weight), while the remaining features are categorical, which in turn include 3 ordinal categorical features, representing sex, educational level, and one disease-specific feature (the jigger infestation classification feature of tungiasis); 2 nominal categorical features (marital status and occupation), representing the unordered categorical data that include marital status occupation; and 10 binary categorical features, represented as “1” or “0” indicating the presence or absence of a given disease-specific symptom. Table S1 in Multimedia Appendix 2 presents the complete dataset information.

**Table 1.** Summarized statistical description of the new dataset used for this study.

Features	Podoconiosis (n=66)	Scabies (n=955)	Tungiasis (n=474)	*P* value
Demographic				
Age (years), mean (SD)	44.68 (13.59)	17.81 (15.17)	21.90 (20.28)	<.001
Sex, n (%)				.104
Female	30 (45.5)	316 (33.1)	154 (32.5)	
Male	36 (54.5)	639 (66.9)	320 (67.5)	
Weight (kg), mean (SD)	52.02 (7.73)	33.31 (15.74)	32.47 (16.70)	<.001
Educational level, mean (SD)	1.26 (0.54)	2.34 (1.38)	2.37 (1.61)	<.001
Clinical				
Disease-specific clinical symptoms	5 (specific symptoms) Slow-enlarging swelling History of barefoot Living podo in endemic area Acute attacks Involving bilateral legs	4 (specific symptoms) Night-worsened itchy lesion Family history of similar condition PPSEs^a^ FWABBP^b^	2 (specific symptoms) Body part infested (hand/leg) Jigger infestation classification	

a Papule, pustule, scratches, and excoriations.

b Finger webs, axilla, breast, buttock, and penis.

### Data Preparation and Preprocessing

#### Data Labeling and Initial Data Preprocessing

In this study, we used IDS in 3 different settings. First, IDS is used with minimal preprocessing operations for developing baseline models. This dataset is used to mimic the original real-world scenario, which is sometimes characterized by incomplete datasets having multiple missing values. As a base dataset, the majority of the essential data preprocessing operations are applied to IDS, while we applied only normalization for FDS and FEFDS, as these datasets were derived from the feature-encoded IDS. Overall, for data preprocessing, we applied data encoding, which mainly included data encoding using label encoding and one-hot encoding methods, including normalization.

For encoding the ordinal categorical features (“sex,” “educational_level,” “JI_classification(#Jig-Inf),” including the target variable “disease_diagnosed”), the Label Encoder method is used. As the remaining 12 features represent nominal categorical data, which include “marital_status,” “occupation,” and 10 disease-specific symptoms, we applied the one-hot encoding method to transform the data under these features. Data normalization was the other preprocessing operation we performed. Accordingly, the numerical features of age (with minimum 2 and maximum 77) and weight (with minimum 8 and maximum 80) are normalized using the “standard scaler” technique to bring these features on the same scale with the other features. This allowed us to maintain a standard normal distribution with zero mean and unit variance within the dataset [29].

#### Handling Structural Missing Data: Feature Engineering

As stated, our new dataset (IDS) contains a total of 11,347 structural null values, which are not random omissions created as a result of the random absence or exclusion of symptoms that are called MCAR or MAR [28]. Over reliance on conventional imputation techniques such as simple imputation or using other methods like mean, median, or multivariate imputation by chained equation (MICE) would introduce biases that could potentially cause both statistical and clinical issues [27,28], having contradictory implications: positively, imputing missing values with “0” values is logically correct, easy to apply, and does not cause any loss of information; while negatively, imputing the missing values with “0” values will introduce hidden danger if a patient develops a secondary (additional) symptom due to a different cause. In our case, for instance, if our models are trained using the fact that “if the values of the ‘Slowenlarging_swelling(feet&legs)’ column are non-zero then, the disease cannot be scabies or tungiasis.” However, such restrictions will affect the generalizability of our models when dealing with patients having mixed symptoms if a patient also has swelling on the feet due to different causes. Therefore, this requires careful analysis and selection of strategies to be used for the imputation, which adds robustness to our models.

Therefore, we created the final dataset by applying a hybrid data encoding strategy: (1) we encoded all symptoms as binary, putting 1 if a given symptom is present (applicable) to a particular disease and putting 0 otherwise; and (2) we introduced a “symptom missingness indicator” parameters, which flags 1 if a given symptom applies to a particular disease and 0 if that symptom does not apply. Therefore, we created a new column called “DISEASE-SYMPTOM_applicable” as a missingness indicator, indicating if that “SYMPTOM” is applicable to the “DISEASE” or not. For instance, since “PPSEs” is a symptom applicable only to scabies and not applicable to podoconiosis or tungiasis, the “PPSEs_applicable” column for all scabies patients is encoded as “1” whether a patient has “PPSEs” or not, while this column is encoded as “0” for podoconiosis and tungiasis patients. The repeated application of this method across 10 disease-specific features adds 10 additional confirmatory columns, which expanded the number of trainable columns of the dataset to 38.

### Experimental Setup

#### Overall Experimental Configurations

This study is conducted based on the stratified *k*-fold cross-validation (CV) method to evaluate possible performance variations resulting from changes in the dataset and ML models and methods. The hold-out method is excluded from the experimental setup due to high-level overfitting recorded during preliminary experiments. Therefore, we conducted extensive experiments using our new dataset in 3 structures (IDS, FDS, and FEFDS) with an 80/20 train-test split based on the stratified 5-fold CV method. We designed 4 separate experimental settings that include (1) baseline training, where IDS (the original dataset) is used with minimal data preprocessing (only data encoding); (2) handling structural missingness, which includes 2 separate training experiments conducted for handling the structural missing values—the first experiment includes training using FDS that applies the simple imputer method, while the next experiment was conducted on FEFDS that applies the symptom applicability indicator flags created after feature engineering—each of these two experiments exactly reflects the other except for the datasets used, aiming to comparatively evaluate performance impacts between the two methods; (3) conditional class weighting; and (4) the hybrid approach, the experiment that harmoniously combines class weighting and the robust dual validation methods, all applied on the dataset created using feature engineering (experiment 3).

#### Conditional Class Weighting

To address the severe class imbalance, we applied a class weighting method. Due to the difference in the internal nature of one of our selected models in accepting the “sample_weight” parameter of the scikit-learn library [30], we conditionally applied the class weighting method. Therefore, for all 7 models that inherently accept the “sample_weight” parameter, these sample weights are automatically computed using the built-in method with the “class_weight” option set to “balanced,” then directly passed to their fit methods. Conversely, as the multilayer perceptron (MLP)–based model does not accept the “sample_weight” parameter, we implemented a fallback strategy based on resampling to replicate the instances of the minority class. In this setting, instances from the training set are resampled with replacement based on probabilities equal to the sample weights computed at the beginning of the class weighting method. We applied this method to facilitate the consistent application of the class weighting method across all the selected models for a complete and fair performance comparison.

#### The Robust Dual Validation Framework

Given the size and distribution of our dataset, using a single *k*-fold (5-fold) split might not help to critically assess the stability and reliability of the models, where the repeated stratified *k*-fold CV method plays vital roles in overcoming performance instability issues [29,31]. Therefore, to establish robust and unbiased performance estimates, we harmoniously applied the repeated and nested stratified CV methods.

##### Repeated Stratified *k*-Fold CV (Outer Loop)

For a severely imbalanced dataset like ours, the distribution of classes across each fold is preserved through stratification. Accordingly, the repeated stratified CV method allows each fold to hold a consistent distribution among the 3 disease classes as a result of the repeated shuffling [32]. Hence, to critically assess the average performance and stability of selected models, we applied the repeated stratified *k*-fold CV method by repeating our previous 5-fold CV loops 10 times to randomly repartition the training set into 5-folds across each repeat while maintaining the class distribution.

##### Nested CV (Inner Loop)

The nested stratified CV is the other ML-based strategy we applied in this study, which is a dual validation strategy consisting of 2 main loops, an inner loop for hyperparameter tuning and an outer loop for validation, collaboratively operating to achieve unbiased performance estimates [30,33]. Accordingly, we implemented the nested CV that uses a stratified *k*-fold loop for the outer loop with 5 folds and another loop for the inner loop with 3 folds. The inner folds are sequentially used by each of the outer folds for selecting the best hyperparameters by running the grid search (GridSearchCV) method on the training portion of each of the outer folds. To ensure unbiased performance estimates, we implemented a procedure to evaluate the best model on the outer test fold, where all the scores on the outer test fold are aggregated.

Overall, in this strategy, we applied the stratification method to maintain the proportional representation of the instances of the minority class (podo) across all training and testing phases. Additionally, to reduce data partitioning bias and evaluate the stability of model prediction results, the complete nested process is repeated 10 times (the repeated stratified *k*-fold CV with *k*=10 is applied) using different random seeds. In this setting, the conditional class balancing method is applied inside the inner loop (inside the inner training folds).

### Model Development

#### Model Selection

**Figure 1.**
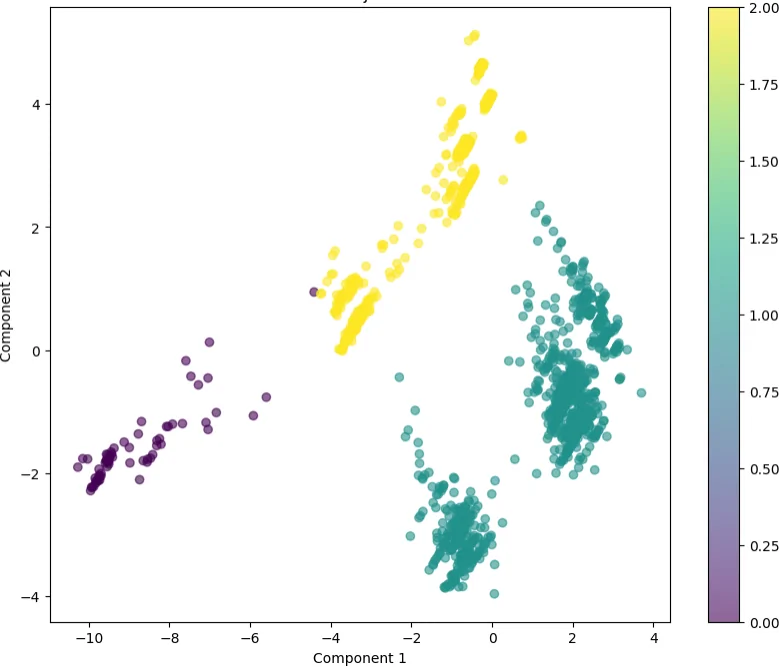
The PCA projection analysis of the features of the datasets. PCA: principal component analysis.

This study uses datasets having 1495 instances with only 38 trainable features, where all the features are mostly linear, as shown by the principal component analysis (PCA) projection in Figure 1.

As shown, the features of the 3 disease classes are highly linear, which shows the tendency of the features to be linearly separable. Hence, given the PCA projection analysis and the datasets to be used, 6 parameters are used to select the models. The parameters used include the model’s ability in handling small-sized datasets, the nature of the data used (mostly linear), the robustness of the model in handling higher-level class imbalances, the robustness of the model in handling data with missing values, higher model performance expected, and model simplicity and interpretability for future deployment of the models on portable devices. Therefore, based on the extended analysis of size, complexity, and overall nature of our datasets, ML models, and the intent of the study, we selected 8 ML models: 3 nontree models, namely naïve Bayes (NB), SVM (with linear kernel), and a simple CNN-based model using MLP; and 5 tree-based models, which include RF (ensemble tree-based model) and 4 boosted tree models of CatBoost, AdaBoost, LightGBM, and XGBoost.

#### Model Evaluation Methods

For this study, we applied a multifaceted evaluation strategy, given the nature of our new dataset. First, the study entirely relies on the stratified *k*-fold CV method, as it allowed us to achieve more robust and lower-variance estimates. Hence, we applied a 5-fold (*k*=5) stratified *k*-fold CV method, as the hold-out method resulted in total overfitting during preliminary experimental trainings. For this study, using the standard accuracy might be misleading given the small-sized dataset used, the simplicity or complexity of models with respect to the dataset used, and the tendency of models to favor the majority class due to the extreme class imbalance [34]. To address this, we used “balanced accuracy,” which is a metric used to reliably evaluate models by giving equal weights for all classes by averaging sensitivity (recall) and specificity (ie, rate of correctly predicted negative classes) of the models [28,29].

The study highly focuses on the *F*_1_-score metric to balance between the precision and recall. The G-mean score, which represents the geometric mean of TPR (true positive rate or recall) and TNR (true negative rate or specificity) [35], is used to evaluate the class-wise accuracy of the models on the 3 disease classes. We used the G-mean along with AUPRC (area under the precision-recall curve) as these metrics are not affected by class imbalances [36], where the AUPRC metric is used to robustly assess the models’ predictive performance on the minority class. To evaluate the models’ class-wise separation performance, the ROC AUC metrics are computed based on the one-vs-one and one-vs-rest classification approaches. Apart from the macro average scores, the class-specific performance of each model is also evaluated to assess how well each model performs on each class, given the severe class imbalance. Hence, the class-specific precision (positive predictive value), recall (sensitivity), specificity (true negative rate), *F*_1_-score, and NPV (negative predictive value) are used to individually assess the models on each class. Confusion matrices are heavily used to compute and visualize the class-specific scores for each model across all experimental settings. Further, we used the feature importance (FI) scores of each model to evaluate model performance and stability, monitor overfitting due to imbalance, include clinical interpretability, and identify noise and redundant features in the dataset. To apply the FI evaluation, we implemented 3 different but complementary techniques that include using built-in importance for the tree-based models, absolute coefficient magnitude for the linear model (SVM), and permutation importance for MLP and NB. Finally, based on the detailed evaluation of the models’ performance, we conducted deeper analysis using descriptive statistical metrics, which include computing average or mean (μ) values, computing and evaluating numerical variations or differences (Δ) of models’ performance, and analyzing performance stability of the models using SD or σ, across the different experimental settings.

## Results

For this study, we conducted the training experiments based on 4 logical experimental settings, as presented by the results in the following subsection.

### Baseline Training

During the first training using IDS based on the 5-fold CV method, all the selected nontree models (NB, SVM, and MLP) and AdaBoost (tree-based model) failed to train due to the null values in the dataset and terminated the training with error messages. These results verified that these 3 nontree and AdaBoost models were highly sensitive to missing values [37], due to the models’ inherent mathematical paradigms of reliance on the availability of complete data compared to the tree-based models [38]. Accordingly, the 4 tree-based models—RF, CatBoost, LightGBM, and XGBoost—were able to classify the diseases with evaluation results, as presented in Table 2.

**Table 2.** Overall model training results on the initial dataset using the *k*-fold cross-validation method.

Model	Accuracy		Macro average						
	Overall accuracy	Balanced accuracy	Precision	Recall	*F*_1_-score	G-mean	ROC-AUC^a^ (OVO^b^)	ROC-AUC (OVR^c^)	AUPRC^d^
Random forest	1.0	1.0	1.0	1.0	1.0	1.0	1.0	1.0	1.0
CatBoost^e^	1.0	1.0	1.0	1.0	1.0	1.0	1.0	1.0	1.0
XGBoost^f^	1.0	1.0	1.0	1.0	1.0	1.0	1.0	1.0	1.0
LightGBM^g^	1.0	1.0	1.0	1.0	1.0	1.0	1.0	1.0	1.0

a ROC-AUC: area under the receiver operating characteristic curve.

b OVO: one-vs-one.

c OVR: one-vs-rest.

d AUPRC: area under the precision-recall curve.

e CatBoost: categorical boosting.

f XGBoost: extreme gradient boosting.

g LightGBM: light gradient boosting machine.

As shown in Table 2, all the 4 models achieved perfect scores (1.0) in accuracy and across all the macro average metrics of precision, recall, *F*_1_-score, G-mean, and ROC-AUC. Class-wise, all these 4 models similarly scored perfect scores (1.0) across all metrics, as shown in Table 3.

**Table 3.** Class-specific results of the 4 trainable models on IDS^a^ using the *k*-fold cross-validation method.

Class	Precision	Recall	*F*_1_-score	TNR^b^	NPV^c^
Podo	1.0	1.0	1.0	1.0	1.0
Scabies	1.0	1.0	1.0	1.0	1.0
Tungiasis	1.0	1.0	1.0	1.0	1.0

a IDS: initial dataset.

b TNR: true negative rate or specificity.

c NPV: negative predictive value.

### Handling Structural Missingness

Table 4 presents overall model performance across the 2 experiments, demonstrating methods for handling missing values.

Starting from the second phase, all 8 models (including NB, SVM, MLP, and AdaBoost that were previously unable to train) were able to run properly as a result of replacing the missing values using the simple imputation method. However, as the null values are structural, an additional experiment demonstrating feature engineering techniques by creating missingness indicator flags was conducted to identify the suitable method for our dataset. Hence, during this experimental setting, we conducted two separate experiments demonstrating the methods we applied to handle structural missingness: (1) experiment A, which was conducted by applying the simple imputation method for the missing disease-specific values, and (2) experiment B, which applied a feature engineering technique and introduced missingness indicator flags for the missing disease-specific values. Table 4 presents the overall results recorded in these 2 trainings. As shown in Table 4, with the use of the simple imputation method, 7 models showed perfect scores (1.0) across all evaluation metrics of balanced accuracy and macro average scores of precision, recall, *F*_1_-score, G-mean, and AUPRC scores. Only MLP scored lower performance compared to the other models by achieving 0.90 in balanced accuracy and macro recall, and 0.77 in precision and *F*_1_-score, while achieving 0.93 G-mean and approximately 1.0 (0.999) AUPRC scores.

**Table 4.** Model performance results when trained using the *k*-fold cross-validation method.

Model	Experiment A: simple imputing						Experiment B: use of missingness indicators					
	Balanced accuracy	Precision	Recall	*F*_1_-score	G-mean	AUPRC^a^	Balanced accuracy	Precision	Recall	*F*_1_-score	G-mean	AUPRC
Naïve Bayes	1.0	1.0	1.0	1.0	1.0	1.0	0.974	0.996	0.974	0.985	0.986	0.972
SVM^b^ (linear)	1.0	1.0	1.0	1.0	1.0	1.0	1.0	1.0	1.0	1.0	1.0	1.0
MLP^c^	0.90	0.77	0.90	0.77	0.93	0.999	0.997	0.956	0.997	0.974	0.997	0.999
Random forest	1.0	1.0	1.0	1.0	1.0	1.0	1.0	1.0	1.0	1.0	1.0	1.0
AdaBoost^d^	1.0	1.0	1.0	1.0	1.0	1.0	1.0	1.0	1.0	1.0	1.0	1.0
CatBoost^e^	1.0	1.0	1.0	1.0	1.0	1.0	1.0	1.0	1.0	1.0	1.0	1.0
XGBoost^f^	1.0	1.0	1.0	1.0	1.0	1.0	1.0	1.0	1.0	1.0	1.0	1.0
LightGBM^g^	1.0	1.0	1.0	1.0	1.0	1.0	1.0	1.0	1.0	1.0	1.0	1.0

a AUPRC: area under the precision-recall curve.

b SVM: support vector machine.

c MLP: multilayer perceptron.

d AdaBoost: adaptive boosting.

e CatBoost: categorical boosting.

f XGBoost: extreme gradient boosting.

g LightGBM: light gradient boosting machine.

Regarding class-specific performance, MLP scored the worst per-class precision of 0.31 for podo, while scoring 1.0 for scabies and tungiasis, as shown in Table 5. MLP also has the lowest *F*_1_-score for podo (0.47), while scoring higher for tungiasis (0.83) and an almost perfect score (0.997) for scabies. However, MLP scored the highest recall and NPV scores for podo compared to scabies and tungiasis.

**Table 5.** Model performance results when trained using the *k*-fold cross-validation method.

Class	NB^a^, SVM^b^, RF^c^, AdaBoost^d^, CatBoost^e^, XGBoost^f^, LightGBM^g^					Per-class scores: MLP^h^				
	Precision	Recall	*F*_1_-score	Specificity	NPV^i^	Precision	Recall	*F*_1_-score	Specificity	NPV
Podo	1.0	1.0	1.0	1.0	1.0	0.31	1.0	0.470	0.899	1.0
Scabies	1.0	1.0	1.0	1.0	1.0	1.0	0.995	0.997	1.0	0.991
Tungiasis	1.0	1.0	1.0	1.0	1.0	1.0	0.705	0.827	1.0	0.879

a NB: Naïve Bayes.

b SVM: support vector machine.

c RF: random forest.

d AdaBoost: adaptive boosting.

e CatBoost: categorical boosting.

f XGBoost: extreme gradient boosting.

g LightGBM: light gradient boosting machine.

h MLP: multilayer perceptron.

i NPV: negative predictive value.

In experiment B, all models scored similar results as the previous simple imputation experiment, except NB and MLP, as shown in Table 4. With the use of the missingness indicators, the NB model achieved declining performance by achieving 0.974 in balanced accuracy, including the 0.97 and 0.985 macro recall and *F*_1_-scores. Conversely, MLP scored significantly improved results compared to the previous experiment with simple imputation, while the remaining 6 models scored perfect scores across all metrics. Class-wise, as shown in Table 6, while MLP achieved improved per-class scores compared to the previous per-class scores (Table 5), NB scored declining performance in the class-specific scores of recall (podo: 0.92), *F*_1_-score (podo: 0.96; tungiasis: 0.995), specificity (tungiasis: 0.995), and NPV (podo: 0.997).

**Table 6.** Model performance results when trained using the *k*-fold cross-validation method.

Class	Naïve Bayes					MLP^a^				
Precision	Recall	*F*_1_-score	Specificity	NPV^b^	Precision	Recall	*F*_1_-score	Specificity	NPV
Podo	1.0	0.92	0.96	1.0	0.997	0.87	1.0	0.93	0.993	1.0
Scabies	1.0	1.0	1.0	1.0	1.0	1.0	0.990	0.995	1.0	0.982
Tungiasis	0.989	1.0	0.995	0.995	1.0	1.0	1.0	1.0	1.0	1.0

a MLP: multilayer perceptron.

b NPV: negative predictive value.

### Class Imbalance Handling

During the third experimental setting, demonstrating the class balancing method, 7 models (including NB) showed stable performance compared to the previous experiment with a missingness indicator. MLP, however, achieved improved results with perfect scores across all metrics, as shown in Table 7.

**Table 7.** Model performance results when trained using the *k*-fold cross-validation method.

Model	Accuracy		Macro average						
	Overall accuracy	Balanced accuracy	Precision	Recall	*F*_1_-score	G-mean	ROC-AUC^a^ (OVO^b^)	ROC-AUC (OVR^c^)	AUPRC^d^
Naïve Bayes	0.997	0.974	0.996	0.974	0.985	0.986	0.981	0.986	0.972
SVM^e^ (linear)	1.0	1.0	1.0	1.0	1.0	1.0	1.0	1.0	1.0
MLP^f^	1.0	1.0	1.0	1.0	1.0	1.0	1.0	1.0	1.0
Random forest	1.0	1.0	1.0	1.0	1.0	1.0	1.0	1.0	1.0
AdaBoost^g^	1.0	1.0	1.0	1.0	1.0	1.0	1.0	1.0	1.0
CatBoost^h^	1.0	1.0	1.0	1.0	1.0	1.0	1.0	1.0	1.0
XGBoost^i^	1.0	1.0	1.0	1.0	1.0	1.0	1.0	1.0	1.0
LightGBM^j^	1.0	1.0	1.0	1.0	1.0	1.0	1.0	1.0	1.0

a ROC-AUC: area under the receiver operating characteristic curve.

b OVO: one-vs-one.

c OVR: one-vs-rest.

d AUPRC: area under the precision-recall curve.

e SVM: support vector machine.

f MLP: multilayer perceptron.

g AdaBoost: adaptive boosting.

h CatBoost: categorical boosting.

i XGBoost: extreme gradient boosting.

j LightGBM: light gradient boosting machine.

In terms of per-class scores, all 7 models (except NB) scored 1.0 across all 5 per-class metrics, as shown in Table 8. NB showed lower per-class scores compared to the other 7 models.

**Table 8.** Model performance results when trained using the *k*-fold cross-validation method.

Class	SVM^a^, RF^b^, AdaBoost^c^, CatBoost^d^, XGBoost^e^, LightGBM^f^, MLP^g^					Naïve Bayes				
	Precision	Recall	*F*_1_-score	Specificity	NPV^h^	Precision	Recall	*F*_1_-score	Specificity	NPV
Podo	1.0	1.0	1.0	1.0	1.0	1.0	0.92	0.96	1.0	0.997
Scabies	1.0	1.0	1.0	1.0	1.0	1.0	1.0	1.0	1.0	1.0
Tungiasis	1.0	1.0	1.0	1.0	1.0	0.989	1.0	0.995	0.995	1.0

a SVM: support vector machine.

b RF: random forest.

c AdaBoost: adaptive boosting.

d CatBoost: categorical boosting.

e XGBoost: extreme gradient boosting.

f LightGBM: light gradient boosting machine.

g MLP: multilayer perceptron.

h NPV: negative predictive value.

### The Harmonized Approach

In this last experimental setting, we applied our proposed harmonized approach that integrates data balancing and robust validation methods, which presents 2 separate evaluation results: first, the performance scored on the test set, and second, the results scored on the nested CV method that incorporates the hyperparameter tuning process. The results in Table 9 present the overall test performance of the models of the final experiment using the hybrid approach. As shown in Table 9, NB and MLP scored similar performance scores of 0.974 in balanced accuracy, with the macro average scores of 0.996, 0.974, 0.985, and 0.986 in precision, recall, *F*_1_-score, and G-mean, respectively. MLP scored higher results in ROC-AUC and AUPRC, while the remaining 6 models scored perfect scores (1.0) across all evaluation metrics.

**Table 9.** Model performance results when trained using the *k*-fold cross-validation method.

Model	Accuracy		Macro average						
	Overall accuracy	Balanced accuracy	Precision	Recall	*F*_1_-score	G-mean	ROC-AUC^a^ (OVO^b^)	ROC-AUC (OVR^c^)	AUPRC^d^
Naïve Bayes	0.997	0.974	0.996	0.974	0.985	0.986	0.981	0.986	0.972
SVM^e^ (linear)	1.0	1.0	1.0	1.0	1.0	1.0	1.0	1.0	1.0
MLP^f^	0.997	0.974	0.996	0.974	0.985	0.986	0.997	1.0	0.999
Random forest	1.0	1.0	1.0	1.0	1.0	1.0	1.0	1.0	1.0
AdaBoost^g^	1.0	1.0	1.0	1.0	1.0	1.0	1.0	1.0	1.0
CatBoost^h^	1.0	1.0	1.0	1.0	1.0	1.0	1.0	1.0	1.0
XGBoost^i^	1.0	1.0	1.0	1.0	1.0	1.0	1.0	1.0	1.0
LightGBM^j^	1.0	1.0	1.0	1.0	1.0	1.0	1.0	1.0	1.0

a ROC-AUC: area under the receiver operating characteristic curve.

b OVO: one-vs-one.

c OVR: one-vs-rest.

d AUPRC: area under the precision-recall curve.

e SVM: support vector machine.

f MLP: multilayer perceptron.

g AdaBoost: adaptive boosting.

h CatBoost: categorical boosting.

i XGBoost: extreme gradient boosting.

j LightGBM: light gradient boosting machine.

Class-wise, 6 models (SVM, RF, AdaBoost, CatBoost, XGBoost, and LightGBM) achieved perfect scores (1.0) across all per-class metrics of precision, recall, *F*_1_-score, specificity, and NPV, as presented in Table 10. Conversely, the MLP and NB models scored exactly the same per-class metrics, scoring the least sensitivity for podoconiosis with a disease-specific recall of 0.92, while scoring almost perfect scores in precision (0.99), specificity (0.995), and *F*_1_-score (0.995) for the tungiasis class.

**Table 10.** Model performance results when trained using the *k*-fold cross-validation method.

Class	Models: SVM^a^, RF^b^, AdaBoost^c^, CatBoost^d^, XGBoost^e^, and LightGBM^f^					Models: NB^g^ and MLP^h^				
	Precision	Recall	*F*_1_-score	Specificity	NPV^i^	Precision	Recall	*F*_1_-score	Specificity	NPV
Podo	1.0	1.0	1.0	1.0	1.0	1.0	0.923	1.0	1.0	0.997
Scabies	1.0	1.0	1.0	1.0	1.0	1.0	1.0	1.0	1.0	1.0
Tungiasis	1.0	1.0	1.0	1.0	1.0	0.989	1.0	0.995	0.995	1.0

a SVM: support vector machine.

b RF: random forest.

c AdaBoost: adaptive boosting.

d CatBoost: categorical boosting.

e XGBoost: extreme gradient boosting.

f LightGBM: light gradient boosting machine.

g NB: Naïve Bayes.

h MLP: multilayer perceptron.

i NPV: negative predictive value.

### Nested CV and Hyperparameter Tuning

Nested CV, the method we applied for hyperparameter tuning and to achieve unbiased performance estimates, resulted in slight deviations compared to the test performance, as summarized in Table 11.

As shown, 5 models scored perfect scores across all metrics with no performance variability (σ=0) on the outer folds. However, 2 models (LightGBM and XGBoost) exhibited slight drops in their average scores of balanced accuracy and macro recall (Δ−0.007, σ=−0.013), and *F*_1_-score (Δ 0.004, σ=−0.007), while the other scores remain stable.

**Table 11.** Summary of models’ performance on the nested cross-validation method, with mean (SD) of key performance metrics.

Model	Balanced accuracy	Recall (macro)	*F*_1_-score (macro)	Average precision	ROC-AUC^a^ (OVO^b^)
Naïve Bayes	0.999 (0.003)	0.999 (0.003)	0.999 (0.003)	0.998 (0.004)	0.999 (0.002)
SVM^c^ (linear)	1.0 (0.0)	1.0 (0.0)	1.0 (0.0)	1.0 (0.0)	1.0 (0.0)
MLP^d^	1.0 (0.0)	1.0 (0.0)	1.0 (0.0)	1.0 (0.0)	1.0 (0.0)
Random forest	1.0 (0.0)	1.0 (0.0)	1.0 (0.0)	1.0 (0.0)	1.0 (0.0)
AdaBoost^e^	1.0 (0.0)	1.0 (0.0)	1.0 (0.0)	1.0 (0.0)	1.0 (0.0)
CatBoost^f^	1.0 (0.0)	1.0 (0.0)	1.0 (0.0)	1.0 (0.0)	1.0 (0.0)
XGBoost^g^	0.993 (0.013)	0.993 (0.013)	0.996 (0.007)	1.0 (0.0)	1.0 (0.0)
LightGBM^h^	0.993 (0.013)	0.993 (0.013)	0.996 (0.008)	1.0 (0.0)	1.0 (0.0)

a ROC-AUC: area under the receiver operating characteristic curve.

b OVO: one-vs-one.

c SVM: support vector machine.

d MLP: multilayer perceptron.

e AdaBoost: adaptive boosting.

f CatBoost: categorical boosting.

g XGBoost: extreme gradient boosting.

h LightGBM: light gradient boosting machine.

### Analysis of Results

#### Overall Performance and Model Stability

**Figure 2.**
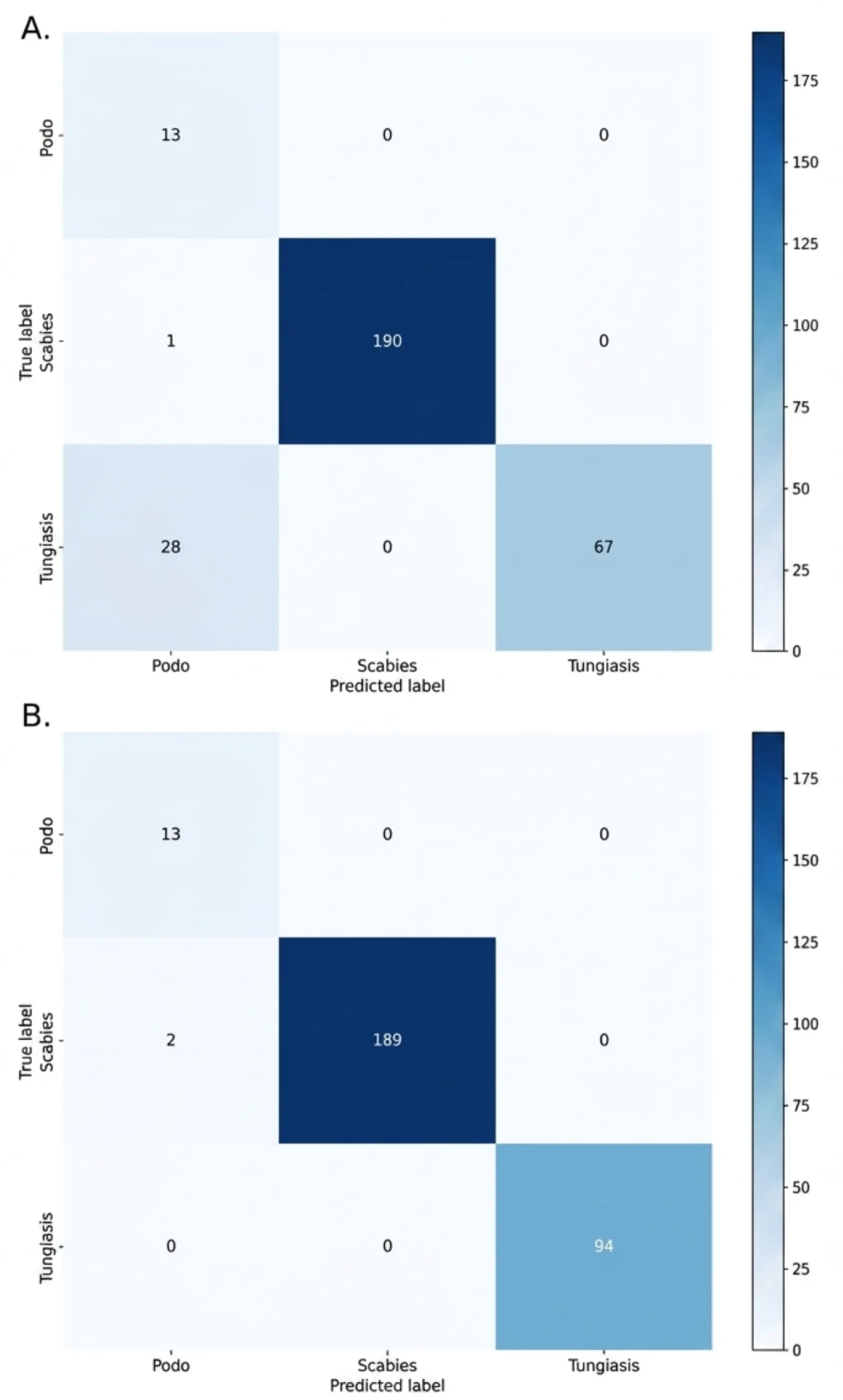
Confusion matrices of MLP showing model performance after the second and third experiments: (A) confusion matrix of MLP after the second experiment with simple imputation and (B) confusion matrix of MLP after the third experiment with feature engineering. MLP: multilayer perceptron.

The second experiment that applied the simple imputation method for the structural missing values introduced the first performance variation, allowing the 4 models (NB, SVM, MLP, and AdaBoost) to have full performance scores. The third experiment brought the next variations, creating 3 divergent performance score groups after the application of disease-specific symptom applicability indicator flags. This experiment resulted in substantial performance changes, particularly to MLP, as evidenced by the model’s absolute improvements of Δ+0.097 in balanced accuracy and macro recall, Δ+0.560 (σ=0.396) in podo precision, Δ+0.460 (σ=0.325) in podo *F*_1_-score, Δ+0.295 (σ=0.209) in tungiasis precision, and Δ+0.173 (σ=0.122) in tungiasis *F*_1_-score. According to the results, all improvements, particularly the class-specific scores, were achieved at a slight expense of the scabies class recall Δ−0.005 (σ=−0.004) and *F*_1_-score Δ−0.002 (σ=−0.001). All these improved scores of MLP underscore that the feature engineering technique was significantly effective in helping the model identify the minority classes. The confusion matrices in Figures 2A and 2B depict all these changes shown by MLP across the 2 experiments. The other 6 models showed all perfect per-class scores with zero misclassification.

Unlike MLP, the generative model NB exhibited overall performance declines of Δ−0.026 in balanced accuracy and macro recall, Δ−0.015 in macro *F*_1_-score, including precision (Δ−0.004) and G-mean (Δ−0.014), including the per-class scores. The remaining 6 models maintained the perfect scores across all metrics. The sample weighting method further improved the performance of MLP with an absolute increase of Δ+0.003 in balanced accuracy, recall, and G-mean, Δ+0.026 in macro *F*_1_-score, and Δ+0.044 in precision, while the remaining 7 models remained consistent.

Overall, across all the experimental phases, 4 models (SVM, RF, CatBoost, and AdaBoost) showed absolute stability of performance, with no performance variation (σ=0.0), while the other 4 models (NB, MLP, XGBoost, and LightGBM) exhibited slight performance variations. Table 12 presents an analysis of models’ performance variance across the 6 major evaluation metrics throughout the 5 experiments. As shown, MLP has the highest overall performance variance in its balanced accuracy and macro recall (σ=0.043), macro *F*_1_-score and precision (σ=0.099), including G-mean (σ=0.03) and macro AUPRC (σ=0.001). Conversely, NB exhibited dropping patterns in balanced accuracy and macro recall (σ=−0.014), macro *F*_1_-score (σ=−0.008), and precision, G-mean, and AUPRC (σ=−0.002, −0.007, and −0.015, respectively). These overall declines were introduced due to the introduction of symptom applicability indicator flags, which created a set of highly correlated features, leading to incompatibility with the model’s probabilistic assumption [39].

According to the results of the nested CV method (Table 12), LightGBM and XGBoost exceptionally exhibited marginal performance drops. These 2 models dropped their performance by Δ−0.007 (σ=−0.003) in balanced accuracy and macro recall, Δ−0.004 (σ=−0.002) in *F*_1_-score and G-mean, while showing no change in precision and AUPRC.

**Table 12.** Analysis of models’ performance based on 6 evaluation metrics across 5 experiments.

Model	Balanced accuracy	Macro average				
		Precision	Recall	*F*_1_-score	G-mean	AUPRC^a^
Naïve Bayes	0.014	0.002	0.014	0.008	0.007	0.015
SVM^b^ (linear)	0.0	0.0	0.0	0.0	0.0	0.0
MLP^c^	0.043	0.099	0.043	0.099	0.03	0.001
Random forest	0.0	0.0	0.0	0.0	0.0	0.0
AdaBoost^d^	0.0	0.0	0.0	0.0	0.0	0.0
CatBoost^e^	0.0	0.0	0.0	0.0	0.0	0.0
XGBoost^f^	0.003	0.0	0.003	0.002	0.002	0.0
LightGBM^g^	0.003	0.0	0.003	0.002	0.002	0.0

a AUPRC: area under the precision-recall curve.

b SVM: support vector machine.

c MLP: multilayer perceptron.

d AdaBoost: adaptive boosting.

e CatBoost: categorical boosting.

f XGBoost: extreme gradient boosting.

g LightGBM: light gradient boosting machine.

Similar patterns were seen in the class-wise performance of these 4 models, as shown in Table 13.

MLP has the highest per-class performance variance with σ=0.33, σ=0.26, σ=0.49, and σ=0.39 in podo-precision, *F*_1_-score, specificity, and recall, respectively. These higher variations are caused by the lowest per-class scores achieved during the third experimental training that demonstrates the structural missing values using missingness indicator features. NB exhibited smaller variance in tungiasis precision (σ=−0.006), podo recall (σ=−0.04), podo-*F*_1_-score (σ=−0.023), tungiasis-*F*_1_-score, and specificity (σ=−0.003). However, the 6 models maintained a totally stable per-class performance (σ=0).

**Table 13.** Summary of models’ performance on the nested cross-validation method, with mean (SD) of key performance metrics.

Model and disease class	Per-class metrics				
	Precision	Recall	*F*_1_-score	Specificity	NPV^a^
SVM^b^, RF^c^, AdaBoost^d^, CatBoost^e^, XGBoost^f^, LightGBM^g^					
Podo	0.0	0.0	0.0	0.0	0.0
Scabies	0.0	0.0	0.0	0.0	0.0
Tungiasis	0.0	0.0	0.0	0.0	0.0
Naïve Bayes					
Podo	0.0	0.040	0.023	0.0	0.002
Scabies	0.0	0.0	0.0	0.0	0.0
Tungiasis	0.006	0.0	0.003	0.003	0.0
MLP^h^					
Podo	0.329	0.039	0.255	0.049	0.002
Scabies	0.0	0.005	0.002	0.0	0.009
Tungiasis	0.006	0.147	0.086	0.003	0.061

a NPV: negative predictive value.

b SVM: support vector machine.

c RF: random forest.

d AdaBoost: adaptive boosting.

e CatBoost: categorical boosting.

f XGBoost: extreme gradient boosting.

g LightGBM: light gradient boosting machine.

h MLP: multilayer perceptron.

#### Analysis of Feature Importance

**Figure 3.**
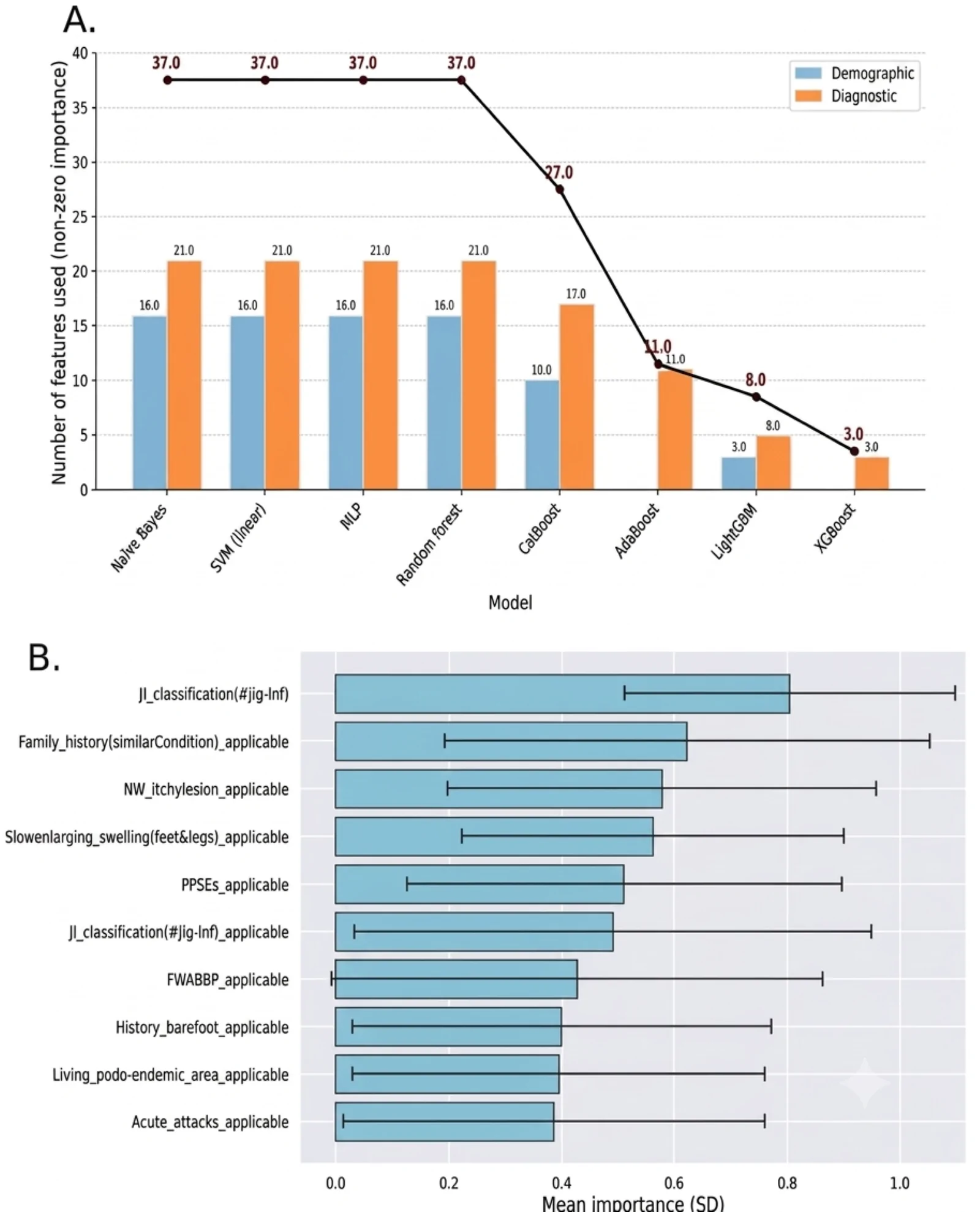
Feature importance evaluation results of the MLP model: (A) overall analysis of the number of features used by each model and (B) top features based on the average importance scores across models. MLP: multilayer perceptron.

While the complete FI scores of the 38 features across 8 models are presented in Table S3 of Multimedia Appendix 2, in this section, we present an overall summary of the FI scores. Accordingly, the FI evaluation results revealed that 4 models (NB, SVM, MLP, and RF) used 37 features out of the 38 trainable features, as shown in Figure 3A.

Regarding individual features used, Figure 3B shows the top 10 features according to their use by the 8 models across all experiments. Accordingly, the jigger infestation classification feature (JI_classification(#jig-inf)) appears to be the top feature having the highest mean FI value. This feature appears in 6 models in their top 5 models list. Model-wise, we conducted deeper analyses of the feature relevance evaluation scores, presented in Table 14, that reveal a diverse set of feature distribution, utilization, and selectivity patterns among models.

As shown, all the 5 tree-based models exhibited a right-skewed FI distribution, which signifies that smaller subsets of the entire feature set are highly relevant, with multiple remaining features being least or nonrelevant [40]. This demonstrated that these 5 tree-based models are highly selective in using features, as evidenced by the feature preference and sparsity ratio scores. Conversely, NB and MLP exhibited a left-skewed FI distribution pattern, highlighting that these models equally designated the majority of the features as highly relevant—MLP assigned the same FI score of 0.665 for 27 features, whereas NB assigned a uniform 1.0 FI score for 36 features. These models have higher nondiscriminative tendencies to identify and ignore less relevant features, as shown by the higher feature preference and lower sparsity ratio scores (Table 14). Finally, SVM is the only model that demonstrated an approximately balanced (symmetric) FI score distribution, as shown by its lower sparsity and information decay scores. Overall, the model-specific FI distributions displayed 3 different distribution trends, which is confirmed by the aggregate summary of FI distribution results (mean 0.311, SD 0.390)—verifying that the overall distribution of the skin NTD diagnostic features is right-skewed. Given the higher mean (μ=0.311) having an SD that exceeds the mean FI score (σ=0.390), which creates higher aggregate variance factors, the right-skewed distribution is naturally expected to be reflected [40].

**Table 14.** Summary of feature importance scores and overall feature usage analysis of the 8 models.

Model	Diagnostic features (n=21)	Demographic features (n=17)	Distribution of FI^a^ score	FI distribution type	Feature preference	Sparsity ratio	Information decay	Information decay type
Naïve Bayes	21	16	Mean 0.974, SD 0.160, range 0-1	Left-skewed	0.9737	0.0263	−0.004	Slow decay
SVM^b^ (linear)	21	16	Mean 0.310, SD 0.334, range 0-1	Approximately symmetric	0.31	0.0526	−0.0284	Slow decay
MLP^c^	21	16	Mean 0.660, SD 0.160, range 0-1	Left-skewed	0.6604	0.0263	−0.0085	Slow decay
Random forest	21	16	Mean 0.232, SD 0.292, range 0-1	Right-skewed	0.2321	0.2105	−0.0239	Slow decay
CatBoost^d^	17	10	Mean 0.069, SD 0.200, range 0-1	Right-skewed	0.0692	0.2895	−0.0096	Slow decay
AdaBoost^e^	11	0	Mean 0.105, SD 0.201, range 0-1	Right-skewed	0.1045	0.7105	−0.013	Slow decay
LightGBM^f^	5	3	Mean 0.061, SD 0.178, range 0-1	Right-skewed	0.061	0.7895	−0.0086	Slow decay
XGBoost^g^	3	0	Mean 0.079, SD 0.270, range 0-1	Right-skewed	0.0789	0.9211	−0.0115	Slow decay
Overall aggregated summary	21	17	Mean 0.311, SD 0.390, range 0-1	Right-skewed	0.311 (0.313)	0.378 (0.347)	−0.0134 (0.0078)	Slow decay

a FI: feature importance.

b SVM: support vector machine.

c MLP: multilayer perceptron.

d CatBoost: categorical boosting.

e AdaBoost: adaptive boosting.

f LightGBM: light gradient boosting machine.

g XGBoost: extreme gradient boosting.

## Discussion

While prior studies proposing AI-assisted diagnosis for skin NTDs primarily focused on image-based approaches, the use of structured clinical skin NTD data, the methodological implications, and enhancement strategies remained to be the major gaps. This preliminary pilot study demonstrated ML-based diagnosis of skin NTDs based on structured patient data, marking a high contribution to the problem domain, through the findings and their implications highlighted hereunder.

### Important Findings

#### Novel Dataset and Diversified Data-Related Challenges

In this study, we crafted a new structured skin NTD clinical dataset, confronted by multifaceted data-related challenges (sample size limitation, severe class imbalance, and lack of full disease representation). We used our novel dataset in 3 different structures with the aim of experimentally analyzing, comparing, and identifying the best-performing ML model for the diagnosis of skin NTDs based on tabular patient data. In achieving the objectives, the study recorded multiple findings with different implications. The use of the initial dataset (having the structural missing values) clearly demonstrated the resiliency of the 4 tree-based models (RF, CatBoost, LightGBM, and XGBoost) to null values due to their built-in mechanisms of handling missing values, which include considering the missing values as a separate group [41,42]. These results underlined the effectiveness of these tree-based architectures to establish robust real-time diagnostic tools, including the scenarios of having incomplete diagnostic data.

#### Methodological Rigor

Given the size and distribution of the new dataset, our study underlined that the selection and utilization of sound ML methods is the key to beat expected generalizability challenges, as demonstrated by the progressive performance gains across experiments. As an initial experimental finding, the Δ+0.097 (10%) and Δ+0.204 (24%) macro recall and *F*_1_-score boosts of MLP validate the adoption of feature engineering as one big initial strategy in addressing and demonstrating our methodological success. Another important finding highlighted that some models, specifically LightGBM and XGBoost, exhibited unreliable performance due to predictive bias, a situation where the standard validation methods (the test CV performance in our case) were pretentiously presenting biased prediction estimates [38,39]. Such performance traps were exposed by the nested CV scores, proving the robustness of the dual CV-based approach in establishing unbiased disease prediction estimates, given the highly constrained dataset used in this study.

Overall, this study highlighted that stable model performances with higher predictive accuracies are achievable through a synergistic integration of multiple ML strategies working in harmony, instead of individual and isolated methods—first, establishing a reliable data preprocessing pipeline followed by a robust validation pipeline incorporating a synchronized implementation of class, all applied on top of the robust data preprocessing pipeline. However, as all these results are achieved against all the odds of data-related constraints, it is mandatory to have further investigations with extended resources to maintain the high achievements.

#### Overall Performance of Models

Based on the overall test performance scores, the majority of the models (except NB and MLP) exhibited consistent performance scores across all metrics. While desirable, perfect scores are not always good signs, given the dataset constraints of this study. Specifically, the perfect scores achieved by the highly feature-selective tree-based models, such as XGBoost, that used only 3 features for disease prediction, raise critical performance concerns. Positively, the perfect scores indicate the maximum disease discriminative power of the model. However, given the fact that the model makes disease predictions based on only 3 features out of 38, and these scores showed declines during the nested CV training, these perfect scores have negative implications, indicating that these scores are fragile and misleading. Such scores indicate that the models are facing the problem of over-optimism in their overall test performance. The use of such models for the diagnosis of diseases using only 3 diagnostic features (3 different symptoms of 3 different diseases) is highly risky and clinically infeasible, as the model would fail if one symptom is missing or misrepresented.

### Implications of Results for Model Selection and Clinical Practices

#### Performance vs Characteristics of Models

According to the FI analysis reports, the diagnostic features have the highest importance for the classification of the skin NTDs and are most widely used by all models, while the demographic features are the least frequently used set of features for the classification of the skin NTDs. These results highlight that models using almost the whole feature set, such as random forest, achieved 100% performance scores across all metrics, while LightGBM (8 features) and XGBoost using only 3 features scored the same 100% performance. These tendencies of the models in using the least proportions of the trainable features could suggest that the models are applying complex architectures and are suffering from overfitting, given the limited dimensionality of our dataset used. Conversely, the tendencies of models to use the highest proportion of the trainable features could also point to the same problem, though such challenges can be alleviated through the use of ML-based strategies, as in the case of this study. Therefore, all these factors suggest that tradeoffs need to be made for the final model selection. Overall performance analysis and FI scores revealed that the 8 models exhibited different behaviors, forming 3 groups of different model characteristics.

#### All-Inclusiveness

Four models (NB, SVM, MLP, and RF) generally fall under this group due to the behavior they commonly share by equally ranking 37 features, while each model has specific determining characteristics. Accordingly, NB showed a different (outlier) behavior due to its invariable selection character that considers all features are equally important, having no interdependency or highly correlated relationship. This high-level feature-inseparability raises an issue, as it is not feasible for 2 or more features to be equally important in diagnosing skin NTDs. For instance, according to the results of our experimental results, a given value of the “educational level” of all patients has never been equally important as the diagnostic features, as confirmed by the dominant diagnostic feature “JI_classification(#Jig-Inf)” and its applicability flag. Further, the introduction of the symptom applicability flags created performance distortions. Both these factors diluted the reliability of the NB models, making them unable to differentiate between symptoms. This, in turn, results in a model inability to differentiate between diseases, which led to perfect scores, where such models cannot be reliable candidates for clinical diagnostics. MLP is the other all-inclusive model with a slightly different feature ranking behavior compared to NB, where 27 features (14 diagnostic and 13 demographic) showed an equal feature rank of 0.665. This depicts invariable ranks (σ=0) among the 27 features, highlighting that the model failed to effectively differentiate disease signal from noise, which also proved that this neural network-based model showed overfitting due to the model’s complex representational ability, given our small-sized tabular dataset. The other 2 models (SVM and RF) showed similar patterns of feature ranking scores with increasing feature sparsity, where the variance in FI scores increased among features.

#### Shortcut Learners

Results showed that 3 of the tree-based models (XGBoost, LightGBM, and AdaBoost) exhibited a “lazy learning” pattern by using a limited number of features (3, 8, and 11 features), while all these 3 models achieved perfect scores. Especially with XGBoost showing absolute feature parsimony (showing high feature selectivity), the perfect scores using only 3 diagnostic features highlight a potential tendency of following shortcut paths by memorizing the feature patterns with the highest performance. Given the dataset constraints and the complexity of these models, this can underscore the plausibility of this shortcut-based (lazy) learning tendency by these 3 models, as exceptionally exposed by the overall performance declines recorded during the nested CV experiment.

#### Final Model Selection: Optimal Classifier

After the extensive analysis of the models’ performance, the study concludes by identifying the model that achieved the optimal performance as the final skin NTD diagnostic model based on structured clinical data. According to the analytic results of the feature preference scores, CatBoost demonstrated the average performance standard compared to all models in our study. Statistically, based on the number of features identified as important (27 features, 71%), the distribution of the features (17 diagnostic and 10 demographic), and performance stability across experimental phases including nested CV, CatBoost showed an optimal separation pattern, unlike the other 2 groups of models. Additionally, its overall internal working logic of ordered boosting (to reduce prediction variances) and inherent categorical feature handling [40], including its fast and stable inference capabilities [43], could also play vital roles in helping the model achieve higher and more stable performance based on an optimal set of features. Therefore, we identified and proposed the CatBoost model as the potential benchmark skin NTD diagnostic model.

### Conclusions

This study developed an ML-based diagnostic model for skin NTDs using a new tabular skin NTD diagnostic dataset (IDS). While the use of FDS (dataset created by applying only data preprocessing) resulted in perfect scores in all models except MLP, the third dataset architecture (created by applying feature engineering on FDS) created a new challenge for NB (due to statistical assumptions of the model), while stabilizing the performance of the other models. These data are used to experimentally analyze, compare, and identify the dataset structure with an optimal set of features, handle structural missing data, control overfitting, and achieve optimal model performance. The class weighting method we used helped in controlling the impact of the severe class imbalance by stabilizing the models’ performance. Though all 6 models scored perfect scores across all metrics (except NB and MLP), the dual CV method proved its robustness, as the nested CV spotted hidden weaknesses in 2 of the tree-based models, XGBoost and LightGBM, showing slight performance declines while these models showed 100% test scores. These two models exhibited a high level of feature parsimony, while NB, SVM (Linear), MLP, and RF were highly feature inclusive. However, CatBoost showed optimal feature ranking and usage patterns. Overall, the boosting tree-based model CatBoost exhibited consistently higher performance, demonstrating optimal feature ranking and usage behavior, which substantiates the robustness of the model. Hence, we recommend further studies with extended resources using the CatBoost model, including RF and SVM as secondary alternatives, to demonstrate reliable diagnostic performance for further deployment.

While this study successfully established a robust ML pipeline as a methodological framework and developed a robust benchmark diagnostic model for skin NTDs, several inherent constraints created restraints to the study, limiting its contribution to its full potential. Data scarcity was the primary limitation of this study, while a severe class imbalance was also a constraint. Other limitations of the study include limited disease representation, inclusion of only one data modality, and specific geographic representation. Therefore, we recommend that further studies be conducted by collecting more patient data from multiple affected and potential areas, with the data being representative of all disease classes that are endemic to Ethiopia. This study also suggests the use of DL-based methods and the inclusion of other patient data (such as images and laboratory results), through proper balancing between diagnostic accuracy and computational expenses, to transform the diagnosis of skin NTDs into higher-level technology-assisted platforms and deliver quality health care services for affected areas, especially for resource-limited areas.

## Supplementary material

10.2196/84966 Multimedia Appendix 1 Distribution of disease classes and missing values in the dataset.

10.2196/84966 Multimedia Appendix 2 Tables presenting statistical analysis, feature importance evaluation scores, and descriptions.

## Abbreviations

AUPRC: area under the precision-recall curve
CL: cutaneous leishmaniasis
CNN: convolutional neural network
CV: cross-validation
DL: deep learning
DTAG: Diagnostic Technical Advisory Group
FDS: final dataset
FEFDS: feature engineering on final dataset
FI: feature importance
IDS: initial dataset
KNN: *k*-nearest neighbors
LightGBM: light gradient boosting model
MAR: missing at random
MCAR: missing completely at random
MDA: mass drug administration
MICE: multivariate imputation by chained equation
ML: machine learning
MLP: multilayer perceptron
NB: naïve Bayes
NPV: negative predictive value
NTD: neglected tropical disease
PCA: principal component analysis
RF: random forest
SVM: support vector machine
TNR: true negative rate
TPR: true positive rate
WHO: World Health Organization
XGBoost: extreme gradient boosting
