# Supplementary material for: Automating Diagnosis of Skin Neglected Tropical Diseases via Patient Metadata through Machine Learning Model with Adaptive Balancing and Dual Cross-Validation: Retrospective Diagnostic Accuracy Study
Source: Online J Public Health Inform. 2026 Aug 21;18:e84966. doi: 10.2196/84966 (PMC13496929; doi:10.2196/84966)
Supplement: Supplementary file 1 [file ojphi-v18-e84966-s001.docx]

### Multimedia Appendix 1. Distribution of disease classes and missing values in the dataset.

**Figure S1.** Distribution of disease classes.

**Figure S2.** Distribution of missing values among disease classes.
