# Supplementary material for: Automating Diagnosis of Skin Neglected Tropical Diseases via Patient Metadata through Machine Learning Model with Adaptive Balancing and Dual Cross-Validation: Retrospective Diagnostic Accuracy Study
Source: Online J Public Health Inform. 2026 Aug 21;18:e84966. doi: 10.2196/84966 (PMC13496929; doi:10.2196/84966)
Supplement: Supplementary file 2 [file ojphi-v18-e84966-s002.docx]

Table S1. Statistical summary of demographic features stratified by the disease class (podo, scabies, tungiasis).

| **IDS** | | | | | | **FDS and FEFDS Primary Features (Without Symptom Applicability Flags)** | | | | |
| --- | --- | --- | --- | --- | --- | --- | --- | --- | --- | --- |
| **Features** | | **Podoconiosis (N=66)** | **Scabies (N=955)** | **Tungiasis (N=474)** | **p-value** |  | **Podoconiosis (N=66)** | **Scabies (N=955)** | **Tungiasis (N=474)** | **p-value** |
| Age | | 44.55 ± 13.43 | 17.81 ± 15.17 | 21.90 ± 20.28 | <0.001 | Age | 44.68 ± 13.59 | 17.81 ± 15.17 | 21.90 ± 20.28 | <0.001 |
| Weight (Kg) | | 52.02 ± 7.73 | 33.31 ± 15.74 | 32.47 ± 16.70 | <0.001 | Weight (Kg) | 52.02 ± 7.73 | 33.31 ± 15.74 | 32.47 ± 16.70 | <0.001 |
| Educational Level | | 1.26 ± 0.54 | 2.34 ± 1.38 | 2.37 ± 1.61 | <0.001 | Educational Level | 1.26 ± 0.54 | 2.34 ± 1.38 | 2.37 ± 1.61 | <0.001 |
| Ji Classification (#Jig-Inf) | | 0.00 ± 0.00 | 0.00 ± 0.00 | 0.30 ± 0.50 | <0.001 | Ji Classification (#Jig-Inf) | 3.00 ± 0.00 | 3.00 ± 0.00 | 0.30 ± 0.50 | <0.001 |
| Sex | - Male (1) | 36 (54.5%) | 639 (66.9%) | 320 (67.5%) | 0.1041 | - Male (1) | 30 (45.5%) | 316 (33.1%) | 154 (32.5%) | 0.1041 |
|  | - Female (0) | 30 (45.5%) | 316 (33.1%) | 154 (32.5%) |  | - Female (0) | 36 (54.5%) | 639 (66.9%) | 320 (67.5%) |  |
| Marital Status: Divorced/Separated | - No (0) | 65 (98.5%) | 955 (100.0%) | 470 (99.2%) | 0.008 | - No (0) | 13 (19.7%) | 726 (76.0%) | 320 (67.5%) | <0.001 |
|  | - Yes (1) | 1 (1.5%) | 0 (0.0%) | 4 (0.8%) |  | - Yes (1) | 53 (80.3%) | 229 (24.0%) | 154 (32.5%) |  |
| Marital Status: Married | - No (0) | 13 (19.7%) | 726 (76.0%) | 320 (67.5%) | <0.001 | - Yes (1) | 5 (7.6%) | 724 (75.8%) | 311 (65.6%) | <0.001 |
|  | - Yes (1) | 53 (80.3%) | 229 (24.0%) | 154 (32.5%) |  | - No (0) | 61 (92.4%) | 231 (24.2%) | 163 (34.4%) |  |
| Marital Status: Unmarried | - Yes (1) | 5 (7.6%) | 724 (75.8%) | 311 (65.6%) | <0.001 | - No (0) | 65 (98.5%) | 955 (100.0%) | 470 (99.2%) | 0.008 |
|  | - No (0) | 61 (92.4%) | 231 (24.2%) | 163 (34.4%) |  | - Yes (1) | 1 (1.5%) | 0 (0.0%) | 4 (0.8%) |  |
| Marital Status: Widowed | - No (0) | 59 (89.4%) | 953 (99.8%) | 469 (98.9%) | <0.001 | - No (0) | 59 (89.4%) | 953 (99.8%) | 469 (98.9%) | <0.001 |
|  | - Yes (1) | 7 (10.6%) | 2 (0.2%) | 5 (1.1%) |  | - Yes (1) | 7 (10.6%) | 2 (0.2%) | 5 (1.1%) |  |
| Occupation: Student | - No (0) | 62 (93.9%) | 505 (52.9%) | 357 (75.3%) | <0.001 | - No (0) | 66 (100.0%) | 737 (77.2%) | 309 (65.2%) | <0.001 |
|  | - Yes (1) | 4 (6.1%) | 450 (47.1%) | 117 (24.7%) |  | - Yes (1) | 0 (0.0%) | 218 (22.8%) | 165 (34.8%) |  |
| Occupation: Employed | - No (0) | 66 (100.0%) | 954 (99.9%) | 474 (100.0%) | 0.7536 | - Yes (1) | 4 (6.1%) | 450 (47.1%) | 117 (24.7%) | <0.001 |
|  | - Yes (1) | 0 (0.0%) | 1 (0.1%) | 0 (0.0%) |  | - No (0) | 62 (93.9%) | 505 (52.9%) | 357 (75.3%) |  |
| Occupation: Nojob/Dependant | - Yes (1) | 0 (0.0%) | 247 (25.9%) | 172 (36.3%) | <0.001 | - No (0) | 66 (100.0%) | 954 (99.9%) | 474 (100.0%) | 0.7536 |
|  | - No (0) | 66 (100.0%) | 708 (74.1%) | 302 (63.7%) |  | - Yes (1) | 0 (0.0%) | 1 (0.1%) | 0 (0.0%) |  |
| Occupation: Farmer | - No (0) | 32 (48.5%) | 816 (85.4%) | 367 (77.4%) | <0.001 | - No (0) | 32 (48.5%) | 816 (85.4%) | 367 (77.4%) | <0.001 |
|  | - Yes (1) | 34 (51.5%) | 139 (14.6%) | 107 (22.6%) |  | - Yes (1) | 34 (51.5%) | 139 (14.6%) | 107 (22.6%) |  |
| Occupation: Housewife | - No (0) | 42 (63.6%) | 869 (91.0%) | 422 (89.0%) | <0.001 | - No (0) | 42 (63.6%) | 869 (91.0%) | 422 (89.0%) | <0.001 |
|  | - Yes (1) | 24 (36.4%) | 86 (9.0%) | 52 (11.0%) |  | - Yes (1) | 24 (36.4%) | 86 (9.0%) | 52 (11.0%) |  |
| Occupation: Housewife Farmer | - No (0) | 63 (95.5%) | 948 (99.3%) | 468 (98.7%) | 0.0127 | - No (0) | 63 (95.5%) | 948 (99.3%) | 468 (98.7%) | 0.0127 |
|  | - Yes (1) | 3 (4.5%) | 7 (0.7%) | 6 (1.3%) |  | - Yes (1) | 3 (4.5%) | 7 (0.7%) | 6 (1.3%) |  |
| Occupation: Merchant | - No (0) | 65 (98.5%) | 951 (99.6%) | 473 (99.8%) | 0.2886 | - No (0) | 65 (98.5%) | 951 (99.6%) | 473 (99.8%) | 0.2886 |
|  | - Yes (1) | 1 (1.5%) | 4 (0.4%) | 1 (0.2%) |  | - Yes (1) | 1 (1.5%) | 4 (0.4%) | 1 (0.2%) |  |
| Occupation: Weaving | - No (0) | 64 (97.0%) | 909 (95.2%) | 443 (93.5%) | 0.2752 | - No (0) | 65 (98.5%) | 934 (97.8%) | 463 (97.7%) | 0.9162 |
|  | - Yes (1) | 2 (3.0%) | 46 (4.8%) | 31 (6.5%) |  | - Yes (1) | 1 (1.5%) | 21 (2.2%) | 11 (2.3%) |  |
| Occupation Other |  | 0 (0.0%) | 955 (100.0%) | 474 (100.0%) | <0.001 | - No (0) | 65 (98.5%) | 901 (94.3%) | 447 (94.3%) | 0.3499 |
|  |  | 64 (97.0%) | 0 (0.0%) | 0 (0.0%) |  | - Yes (1) | 1 (1.5%) | 54 (5.7%) | 27 (5.7%) |  |

Table S2. Statistical summary of diagnostic features stratified by the disease class (podo, scabies, tungiasis).

| IDS | | | | | FDS and FEFDS Primary Features (Without Symptom Applicability Flags) | | | | |
| --- | --- | --- | --- | --- | --- | --- | --- | --- | --- |
|  | **Podoconiosis (N=66)** | **Scabies (N=955)** | **Tungiasis (N=474)** | **p-value** |  | **Podoconiosis (N=66)** | **Scabies (N=955)** | **Tungiasis (N=474)** | **p-value** |
| Slow Enlarging Swelling (Feet/Legs) |  |  |  |  | Slow Enlarging Swelling (Feet/Legs) |  |  |  |  |
| **- Unknown** | **0 (0.0%)** | **955 (100.0%)** | **474 (100.0%)** | **<0.001** |  |  |  |  |  |
| - Yes (1.0) | 64 (97.0%) | 0 (0.0%) | 0 (0.0%) |  | -1 | 64 (97.0%) | 0 (0.0%) | 0 (0.0%) | <0.001 |
| - No (0.0) | 2 (3.0%) | 0 (0.0%) | 0 (0.0%) |  | 0 | 2 (3.0%) | 955 (100.0%) | 474 (100.0%) |  |
| History: Barefoot |  |  |  |  | History Barefoot |  |  |  |  |
| **- Unknown** | **0 (0.0%)** | **955 (100.0%)** | **474 (100.0%)** | **<0.001** |  |  |  |  |  |
| - Yes (1.0) | 62 (93.9%) | 0 (0.0%) | 0 (0.0%) |  | 0 | 4 (6.1%) | 955 (100.0%) | 474 (100.0%) | <0.001 |
| - No (0.0) | 4 (6.1%) | 0 (0.0%) | 0 (0.0%) |  | -1 | 62 (93.9%) | 0 (0.0%) | 0 (0.0%) |  |
| Living in Podo-Endemic Area |  |  |  |  | Living Podo-Endemic Area |  |  |  |  |
| **- Unknown** | **0 (0.0%)** | **955 (100.0%)** | **474 (100.0%)** | **<0.001** |  |  |  |  |  |
| - No (0.0) | 22 (33.3%) | 0 (0.0%) | 0 (0.0%) |  | 0 | 22 (33.3%) | 955 (100.0%) | 474 (100.0%) | <0.001 |
| - Yes (1.0) | 44 (66.7%) | 0 (0.0%) | 0 (0.0%) |  | -1 | 44 (66.7%) | 0 (0.0%) | 0 (0.0%) |  |
| Acute Attacks |  |  |  |  | Acute Attacks |  |  |  |  |
| **- Unknown** | **0 (0.0%)** | **955 (100.0%)** | **474 (100.0%)** | **<0.001** |  |  |  |  |  |
| - No (0.0) | 4 (6.1%) | 0 (0.0%) | 0 (0.0%) |  | -1 | 62 (93.9%) | 0 (0.0%) | 0 (0.0%) | <0.001 |
| - Yes (1.0) | 62 (93.9%) | 0 (0.0%) | 0 (0.0%) |  | 0 | 4 (6.1%) | 955 (100.0%) | 474 (100.0%) |  |
| Involving Bilateral |  |  |  |  | Involving Bilateral |  |  |  |  |
| **- Unknown** | **0 (0.0%)** | **955 (100.0%)** | **474 (100.0%)** | **<0.001** |  |  |  |  |  |
| - Yes (1.0) | 63 (95.5%) | 0 (0.0%) | 0 (0.0%) |  | -1 | 63 (95.5%) | 0 (0.0%) | 0 (0.0%) | <0.001 |
| - No (0.0) | 3 (4.5%) | 0 (0.0%) | 0 (0.0%) |  | 0 | 3 (4.5%) | 955 (100.0%) | 474 (100.0%) |  |
| Nw Itchy Lesion |  |  |  |  | Nw Itchylesion |  |  |  |  |
| - Yes (1.0) | 0 (0.0%) | 953 (99.8%) | 0 (0.0%) | <0.001 |  |  |  |  |  |
| **- Unknown** | **66 (100.0%)** | **0 (0.0%)** | **474 (100.0%)** |  | 0 | 66 (100.0%) | 2 (0.2%) | 474 (100.0%) | <0.001 |
| - No (0.0) | 0 (0.0%) | 2 (0.2%) | 0 (0.0%) |  | -1 | 0 (0.0%) | 953 (99.8%) | 0 (0.0%) |  |
| Family History |  |  |  |  | Family History (Similar Condition) |  |  |  |  |
| - Yes (1.0) | 0 (0.0%) | 912 (95.5%) | 0 (0.0%) | <0.001 |  |  |  |  |  |
| - No (0.0) | 0 (0.0%) | 43 (4.5%) | 0 (0.0%) |  | 0 | 66 (100.0%) | 43 (4.5%) | 474 (100.0%) | <0.001 |
| **- Unknown** | **66 (100.0%)** | **0 (0.0%)** | **474 (100.0%)** |  | **-1** | **0 (0.0%)** | **912 (95.5%)** | **0 (0.0%)** |  |
| Ppses |  |  |  |  | Ppses |  |  |  |  |
| - No (0.0) | 0 (0.0%) | 418 (43.8%) | 0 (0.0%) | <0.001 |  |  |  |  |  |
| - Yes (1.0) | 0 (0.0%) | 537 (56.2%) | 0 (0.0%) |  | 0 | 66 (100.0%) | 418 (43.8%) | 474 (100.0%) | <0.001 |
| **- Unknown** | **66 (100.0%)** | **0 (0.0%)** | **474 (100.0%)** |  | -1 | 0 (0.0%) | 537 (56.2%) | 0 (0.0%) |  |
| Fwabbp |  |  |  |  | Fwabbp |  |  |  |  |
| - No (0.0) | 0 (0.0%) | 102 (10.7%) | 0 (0.0%) | <0.001 |  |  |  |  |  |
| - Yes (1.0) | 0 (0.0%) | 853 (89.3%) | 0 (0.0%) |  | 0 | 66 (100.0%) | 102 (10.7%) | 474 (100.0%) | <0.001 |
| **- Unknown** | **66 (100.0%)** | **0 (0.0%)** | **474 (100.0%)** |  | -1 | 0 (0.0%) | 853 (89.3%) | 0 (0.0%) |  |
| Body Part Infested |  |  |  |  | Bodypart Infested |  |  |  |  |
| **- Unknown** | **66 (100.0%)** | **955 (100.0%)** | **0 (0.0%)** | **<0.001** |  |  |  |  |  |
| - Yes (1.0) | 0 (0.0%) | 0 (0.0%) | 468 (98.7%) |  | -1 | 66 (100.0%) | 44 (4.6%) | 468 (98.7%) | <0.001 |
| - No (0.0) | 0 (0.0%) | 0 (0.0%) | 6 (1.3%) |  | 0 | 0 (0.0%) | 911 (95.4%) | 6 (1.3%) |  |

Table S3. Statistical summary of diagnostic features, symptom applicability indicator flags that specifically belong to the FEFDS dataset created after feature engineering.

| FEFDS Symptom Applicability Flags | | | | |
| --- | --- | --- | --- | --- |
|  | **Podoconiosis (N=66)** | **Scabies (N=955)** | **Tungiasis (N=474)** | **p-value** |
| Slow Enlarging Swelling Applicable |  |  |  |  |
| - Yes (1.0) | 66 (100.0%) | 0 (0.0%) | 0 (0.0%) | <0.001 |
| - No (0.0) | 0 (0.0%) | 955 (100.0%) | 474 (100.0%) |  |
| History Barefoot Applicable |  |  |  |  |
| - Yes (1.0) | 66 (100.0%) | 0 (0.0%) | 0 (0.0%) | <0.001 |
| - No (0.0) | 0 (0.0%) | 955 (100.0%) | 474 (100.0%) |  |
| Living Podo-Endemic Area Applicable | |  |  |  |
| - Yes (1.0) | 66 (100.0%) | 0 (0.0%) | 0 (0.0%) | <0.001 |
| - No (0.0) | 0 (0.0%) | 955 (100.0%) | 474 (100.0%) |  |
| Acute Attacks Applicable |  |  |  |  |
| - Yes (1.0) | 66 (100.0%) | 0 (0.0%) | 0 (0.0%) | <0.001 |
| - No (0.0) | 0 (0.0%) | 955 (100.0%) | 474 (100.0%) |  |
| Involving Bilateral Applicable |  |  |  |  |
| - Yes (1.0) | 66 (100.0%) | 0 (0.0%) | 0 (0.0%) | <0.001 |
| - No (0.0) | 0 (0.0%) | 955 (100.0%) | 474 (100.0%) |  |
| Nw Itchylesion Applicable |  |  |  |  |
| - No (0) | 66 (100.0%) | 0 (0.0%) | 474 (100.0%) | <0.001 |
| - Yes (1) | 0 (0.0%) | 955 (100.0%) | 0 (0.0%) |  |
| Family History Applicable |  |  |  |  |
| - No (0) | 66 (100.0%) | 0 (0.0%) | 474 (100.0%) | <0.001 |
| - Yes (1) | 0 (0.0%) | 955 (100.0%) | 0 (0.0%) |  |
| Ppses Applicable |  |  |  |  |
| - No (0) | 66 (100.0%) | 0 (0.0%) | 380 (80.2%) | <0.001 |
| - Yes (1) | 0 (0.0%) | 955 (100.0%) | 94 (19.8%) |  |
| Fwabbp Applicable |  |  |  |  |
| - No (0) | 66 (100.0%) | 0 (0.0%) | 474 (100.0%) | <0.001 |
| - Yes (1) | 0 (0.0%) | 955 (100.0%) | 0 (0.0%) |  |
| Ji Classification Applicable |  |  |  |  |
| - No (0) | 66 (100.0%) | 955 (100.0%) | 0 (0.0%) | <0.001 |
| - Yes (1) | 0 (0.0%) | 0 (0.0%) | 474 (100.0%) |  |

Table S4. Overall feature importance (FI) scores assigned for each of the 38 features and overall statistical analysis of the FI scores.

| **#** | **Features** | **Feature-Category** | **NB** | **SVM (Linear)** | **MLP** | **RF** | **AdaBoost** | **CatBoost** | **LGBoost** | **XGBoost** |  | **Top FI** | **Top Scoring Model** | **Least FI** | **Least Scoring Model** | **Average (µ)** | **FI Variability (σ)** | **CV** |
| --- | --- | --- | --- | --- | --- | --- | --- | --- | --- | --- | --- | --- | --- | --- | --- | --- | --- | --- |
| **1** | **JI_classification(#Jig-Inf)_applicable** | **Diagnostic** | 1.0 | 1.000 | 0.665 | 0.913 | 0.337 | 0.003 | 0.0 | 0.0 |  | **1.0** | **SVM (Linear)** | **0.0** | **LGBoost** | **0.490** | **0.458** | **0.936** |
| **2** | **family_history(similarCondition)_applicable** | **Diagnostic** | 1.0 | 0.956 | 0.923 | 0.652 | 0.440 | 1.000 | 0.0 | 0.0 |  | **1.0** | **CatBoost** | **0.0** | **LGBoost** | **0.621** | **0.430** | **0.692** |
| **3** | **PPSEs_applicable** | **Diagnostic** | 1.0 | 0.956 | 0.665 | 0.684 | 0.360 | 0.420 | 0.0 | 0.0 |  | **1.0** | **NB** | **0.0** | **LGBoost** | **0.511** | **0.386** | **0.756** |
| **4** | **FWABBP_applicable** | **Diagnostic** | 1.0 | 0.956 | 0.665 | 0.639 | 0.160 | 0.002 | 0.0 | 0.0 |  | **1.0** | **NB** | **0.0** | **LGBoost** | **0.428** | **0.435** | **1.018** |
| **5** | **NW_itchylesion_applicable** | **Diagnostic** | 1.0 | 0.956 | 0.665 | 0.360 | 0.351 | 0.017 | 0.268 | 1.0 |  | **1.0** | **XGBoost** | **0.0** | **CatBoost** | **0.577** | **0.381** | **0.660** |
| **6** | **Slowenlarging_swelling(feet&legs)_applicable** | **Diagnostic** | 1.0 | 0.591 | 0.674 | 0.560 | 0.240 | 0.036 | 0.390 | 1.000 |  | **1.0** | **NB** | **0.0** | **CatBoost** | **0.561** | **0.340** | **0.605** |
| **7** | **history_barefoot_applicable** | **Diagnostic** | 1.0 | 0.591 | 0.665 | 0.590 | 0.320 | 0.027 | 0.0 | 0.0 |  | **1.0** | **NB** | **0.0** | **LGBoost** | **0.399** | **0.372** | **0.932** |
| **8** | **acute_attacks_applicable** | **Diagnostic** | 1.0 | 0.591 | 0.665 | 0.586 | 0.200 | 0.047 | 0.0 | 0.0 |  | **1.0** | **NB** | **0.0** | **LGBoost** | **0.386** | **0.375** | **0.971** |
| **9** | **living_podo-endemic_area_applicable** | **Diagnostic** | 1.0 | 0.591 | 0.665 | 0.572 | 0.244 | 0.087 | 0.0 | 0.0 |  | **1.0** | **NB** | **0.0** | **LGBoost** | **0.395** | **0.366** | **0.927** |
| **10** | **involving_bilateral_applicable** | **Diagnostic** | 1.0 | 0.591 | 0.665 | 0.401 | 0.320 | 0.006 | 0.0 | 0.0 |  | **1.0** | **NB** | **0.0** | **LGBoost** | **0.373** | **0.367** | **0.984** |
| **11** | **NW_itchylesion** | **Diagnostic** | 1.0 | 0.589 | 0.665 | 0.415 | 0.0 | 0.013 | 0.122 | 0.0 |  | **1.0** | **NB** | **0.0** | **AdaBoost** | **0.350** | **0.377** | **1.075** |
| **12** | **acute_attacks** | **Diagnostic** | 1.0 | 0.485 | 0.665 | 0.167 | 0.0 | 0.038 | 0.0 | 0.0 |  | **1.0** | **NB** | **0.0** | **AdaBoost** | **0.294** | **0.380** | **1.293** |
| **13** | **involving_bilateral** | **Diagnostic** | 1.0 | 0.480 | 0.665 | 0.165 | 0.0 | 0.005 | 0.0 | 0.0 |  | **1.0** | **NB** | **0.0** | **AdaBoost** | **0.289** | **0.383** | **1.325** |
| **14** | **JI_classification(#Jig-Inf)** | **Diagnostic** | 1.0 | 0.467 | 1.000 | 1.000 | 1.000 | 0.698 | 0.268 | 1.000 |  | **1.0** | **AdaBoost** | **0.3** | **LGBoost** | **0.804** | **0.294** | **0.365** |
| **15** | **family_history(similarCondition)** | **Diagnostic** | 1.0 | 0.386 | 0.665 | 0.085 | 0.0 | 0.0 | 0.0 | 0.0 |  | **1.0** | **NB** | **0.0** | **AdaBoost** | **0.267** | **0.383** | **1.436** |
| **16** | **bodyPart_infested** | **Diagnostic** | 1.0 | 0.311 | 0.713 | 0.235 | 0.0 | 0.0 | 0.0 | 0.0 |  | **1.0** | **NB** | **0.0** | **AdaBoost** | **0.282** | **0.382** | **1.353** |
| **17** | **history_barefoot** | **Diagnostic** | 1.0 | 0.246 | 0.684 | 0.208 | 0.0 | 0.002 | 0.0 | 0.0 |  | **1.0** | **NB** | **0.0** | **AdaBoost** | **0.267** | **0.378** | **1.413** |
| **18** | **Slowenlarging_swelling(feet&legs)** | **Diagnostic** | 1.0 | 0.241 | 0.665 | 0.439 | 0.0 | 0.169 | 0.171 | 0.0 |  | **1.0** | **NB** | **0.0** | **AdaBoost** | **0.336** | **0.348** | **1.038** |
| **19** | **FWABBP** | **Diagnostic** | 1.0 | 0.153 | 0.732 | 0.000 | 0.0 | 0.004 | 0.0 | 0.0 |  | **1.0** | **NB** | **0.0** | **AdaBoost** | **0.236** | **0.399** | **1.689** |
| **20** | **PPSEs** | **Diagnostic** | 1.0 | 0.034 | 0.665 | 0.045 | 0.0 | 0.0 | 0.0 | 0.0 |  | **1.0** | **NB** | **0.0** | **AdaBoost** | **0.218** | **0.390** | **1.789** |
| **21** | **living_podo-endemic_area** | **Diagnostic** | 1.0 | 0.001 | 0.674 | 0.046 | 0.0 | 0.0 | 0.0 | 0.0 |  | **1.0** | **NB** | **0.0** | **AdaBoost** | **0.215** | **0.394** | **1.831** |
| **22** | **age** | **Demographic** | 1.0 | 0.203 | 0.665 | 0.022 | 0.0 | 0.015 | 1.000 | 0.0 |  | **1.0** | **LGBoost** | **0.0** | **AdaBoost** | **0.363** | **0.452** | **1.245** |
| **23** | **weight(Kg)** | **Demographic** | 1.0 | 0.121 | 0.665 | 0.018 | 0.0 | 0.004 | 0.073 | 0.0 |  | **1.0** | **NB** | **0.0** | **AdaBoost** | **0.235** | **0.382** | **1.623** |
| **24** | **educational_level** | **Demographic** | 1.0 | 0.061 | 0.665 | 0.010 | 0.0 | 0.005 | 0.0 | 0.0 |  | **1.0** | **NB** | **0.0** | **AdaBoost** | **0.218** | **0.390** | **1.794** |
| **25** | **occupation_Farmer** | **Demographic** | 1.0 | 0.050 | 0.665 | 0.000 | 0.0 | 8.03E-06 | 0.0 | 0.0 |  | **1.0** | **NB** | **0.0** | **AdaBoost** | **0.214** | **0.392** | **1.829** |
| **26** | **marital_status_Unmarried** | **Demographic** | 1.0 | 0.044 | 0.665 | 0.003 | 0.0 | 0.007 | 0.0 | 0.0 |  | **1.0** | **NB** | **0.0** | **AdaBoost** | **0.215** | **0.392** | **1.824** |
| **27** | **marital_status_Married** | **Demographic** | 1.0 | 0.043 | 0.665 | 0.000 | 0.0 | 0.008 | 0.0 | 0.0 |  | **1.0** | **NB** | **0.0** | **AdaBoost** | **0.214** | **0.392** | **1.828** |
| **28** | **occupation_Child/dependant** | **Demographic** | 1.0 | 0.032 | 0.665 | 0.000 | 0.0 | 0.001 | 0.0 | 0.0 |  | **1.0** | **NB** | **0.0** | **AdaBoost** | **0.212** | **0.393** | **1.853** |
| **29** | **sex** | **Demographic** | 1.0 | 0.028 | 0.665 | 0.000 | 0.0 | 0.0 | 0.0 | 0.0 |  | **1.0** | **NB** | **0.0** | **AdaBoost** | **0.212** | **0.394** | **1.860** |
| **30** | **occupation_Student** | **Demographic** | 1.0 | 0.015 | 0.933 | 0.002 | 0.0 | 0.0 | 0.024 | 0.0 |  | **1.0** | **NB** | **0.0** | **AdaBoost** | **0.247** | **0.445** | **1.802** |
| **31** | **occupation_Other** | **Demographic** | 1.0 | 0.005 | 0.144 | 0.000 | 0.0 | 0.0 | 0.0 | 0.0 |  | **1.0** | **NB** | **0.0** | **AdaBoost** | **0.144** | **0.350** | **2.433** |
| **32** | **occupation_HouseWife** | **Demographic** | 1.0 | 0.004 | 0.665 | 0.000 | 0.0 | 0.005 | 0.0 | 0.0 |  | **1.0** | **NB** | **0.0** | **AdaBoost** | **0.209** | **0.395** | **1.887** |
| **33** | **marital_status_Widowed** | **Demographic** | 1.0 | 0.003 | 0.674 | 0.001 | 0.0 | 0.012 | 0.0 | 0.0 |  | **1.0** | **NB** | **0.0** | **AdaBoost** | **0.211** | **0.396** | **1.874** |
| **34** | **occupation_weaving** | **Demographic** | 1.0 | 0.001 | 0.665 | 0.000 | 0.0 | 0.0 | 0.0 | 0.0 |  | **1.0** | **NB** | **0.0** | **AdaBoost** | **0.208** | **0.396** | **1.900** |
| **35** | **occupation_merchant** | **Demographic** | 1.0 | 0.000 | 0.665 | 0.000 | 0.0 | 0.0 | 0.0 | 0.0 |  | **1.0** | **NB** | **0.0** | **AdaBoost** | **0.208** | **0.396** | **1.901** |
| **36** | **occupation_HouseWifeFarmer** | **Demographic** | 1.0 | 0.000 | 0.665 | 0.000 | 0.0 | 0.0 | 0.0 | 0.0 |  | **1.0** | **NB** | **0.0** | **AdaBoost** | **0.208** | **0.396** | **1.901** |
| **37** | **marital_status_Divorced** | **Demographic** | 0.0 | 1.70E-18 | 0.0 | 0.0 | 0.0 | 0.001 | 0.0 | 0.0 |  | **0.0** | **CatBoost** | **0.0** | **NB** | **0.000** | **0.000** | **2.828** |
| **38** | **occupation_Employed** | **Demographic** | 1.0 | 0.0 | 0.665 | 3.33E-16 | 0.0 | 0.0 | 0.0 | 0.0 |  | **1.0** | **NB** | **0.0** | **SVM (Linear)** | **0.208** | **0.396** | **1.901** |
|  |  |  |  |  |  |  |  |  |  |  |  |  |  |  |  |  |  |  |
|  | **Summary of FI Across Features** | **Model** | **NB** | **SVM (Linear)** | **MLP** | **RF** | **AdaBoost** | **CatBoost** | **LGBoost** | **XGBoost** |  |  |  |  |  |  |  |  |
|  |  | **Total Feature Used** | **37** | **37** | **37** | **37** | **11** | **27** | **8** | **3** |  |  |  |  |  |  |  |  |
|  |  | **Max. FI Score** | **1.0** | **1.0** | **1.0** | **1.0** | **1.0** | **1.0** | **1.0** | **1.0** |  |  |  |  |  |  |  |  |
|  |  | **Min. FI Score** | **0.0** | **0.0** | **0.0** | **0.0** | **0.0** | **0.0** | **0.0** | **0.0** |  |  |  |  |  |  |  |  |
|  |  | **Average FI Score (µ)** | **0.974** | **0.310** | **0.660** | **0.232** | **0.105** | **0.069** | **0.061** | **0.079** |  |  |  |  |  |  |  |  |
|  |  | **FI Variability (σ)** | **0.162** | **0.338** | **0.162** | **0.296** | **0.204** | **0.203** | **0.180** | **0.273** |  |  |  |  |  |  |  |  |
|  |  | **CV** | **0.167** | **1.091** | **0.245** | **1.275** | **1.947** | **2.931** | **2.960** | **3.461** |  |  |  |  |  |  |  |  |
